# Size control of ropivacaine nano/micro-particles by soft-coating with peptide nanosheets for long-acting analgesia

**DOI:** 10.7150/thno.93322

**Published:** 2024-04-15

**Authors:** Jing Liu, Weiwei Wu, Fei Peng, Deying Gong, Yi Kang, Yujun Zhang, Congyan Liu, Yuncheng Li, Guoyan Zhao, Feng Qiu, Wensheng Zhang

**Affiliations:** 1 Department of Anesthesiology, West China Hospital, Sichuan University, China.; 2 Laboratory of Anesthesia and Critical Care Medicine, National-Local Joint Engineering Research Centre of Translational Medicine of Anesthesiology, West China Hospital, Sichuan University, China.

**Keywords:** long-acting analgesia, particle size, ropivacaine, slow-release, self-assembling peptides

## Abstract

**Rationale:** To meet the need of long-acting analgesia in postoperative pain management, slow-releasing formulations of local anesthetics (LAs) have been extensively investigated. However, challenges still remain in obtaining such formulations in a facile and cost-effective way, and a mechanism for controlling the release rate to achieve an optimal duration is still missing.

**Methods:** In this study, nanosheets formed by a self-assembling peptide were used to encapsulate ropivacaine in a soft-coating manner. By adjusting the ratio between the peptide and ropivacaine, ropivacaine particles with different size were prepared. Releasing profile of particles with different size were studied *in vitro* and *in vivo*. The influence of particle size and ropivacaine concentration on effective duration and toxicity were evaluated in rat models.

**Results:** Our results showed that drug release rate became slower as the particle size increased, with particles of medium size (2.96 ± 0.04 μm) exhibiting a moderate release rate and generating an optimal anesthetic duration. Based on this size, formulations at different ropivacaine concentrations generated anesthetic effect with different durations in rat sciatic nerve block model, with the 6% formulation generated anesthetic duration of over 35 h. Long-acting analgesia up to 48 h of this formulation was also confirmed in a rat total knee arthroplasty model.

**Conclusion:** This study provided a facile strategy to prepare LA particles of different size and revealed the relationship between particle size, release rate and anesthetic duration, which provided both technical and theoretical supports for developing long-acting LA formulations with promising clinical application.

## Introduction

Ever since the popularization of surgical intervention as an effective clinical treatment, postoperative pain has long been an inevitable issue to be addressed. Although opioids have shown strong and reliable analgesic efficacy, associated side effects and pervading opioid crisis have raised an urgent need to reduce their consumption 1, 2. In this regard, local anesthetics (LAs) have become a popular choice for the management of postoperative pain due to their fast onset, low toxicity and non-addictiveness 3-5. However, the analgesic effect of traditional LA formulations such as ropivacaine hydrochloride (RH) and bupivacaine hydrochloride could only last for several hours, which is far from sufficiently suppressing postoperative pain generally lasting for 2-3 days 6.

To prolong the analgesic duration of traditional LAs, many slow-releasing LA formulations have been developed based on various types of materials including polymeric microspheres 7-9, liposomes 10, 11, hydrogels 12-14, peptides 15 and their hybrid systems 16, 17. Unfortunately, before the translation to clinical application, most formulations developed in laboratory are still facing many challenges including complicated preparation process, low drug-loading capacity and biocompatibility issue of the carrier materials. As a novel type of carrier materials for advanced drug delivery systems, self-assembling peptides (SAPs) composed of natural amino acids have attracted more and more attention for their excellent biocompatibility and biodegradability 18-20. In our previous studies, SAPs were used to form nanoparticle complexes with soluble LA hydrochlorides, which could induce the bottom-up formation of slow-releasing LA nano/micro-crystals with prolonged analgesic duration and considerable safety 21-23.

Inspired by these slow-releasing LA nano/micro-crystals, we realized that LAs in their water-insoluble base form could be used as an innate slow-releasing drug deposit. However, an effective slow-releasing formulation is not merely LAs in their base form. Actually, although the formation of LA crystals has also been noticed in some clinical practices, these crystals have been found to be unfavorable for the anesthetic effect, possibly because of their inefficient drug release 24, 25. It is quite clear that to achieve long-acting anesthesia, releasing profile of the formulations should be deliberately controlled within a range between the effective and toxic thresholds (Figure S1). Interestingly, several recent studies have proved that the size of drug particles was an important parameter affecting their release rate and biodistribution, suggesting a simple strategy to control the release rate 26-28. And for controlling the size of drug particles, top-down strategies provided a more straightforward way compared with the bottom-up crystallization processes mentioned above 29, 30.

**Figure 1:**
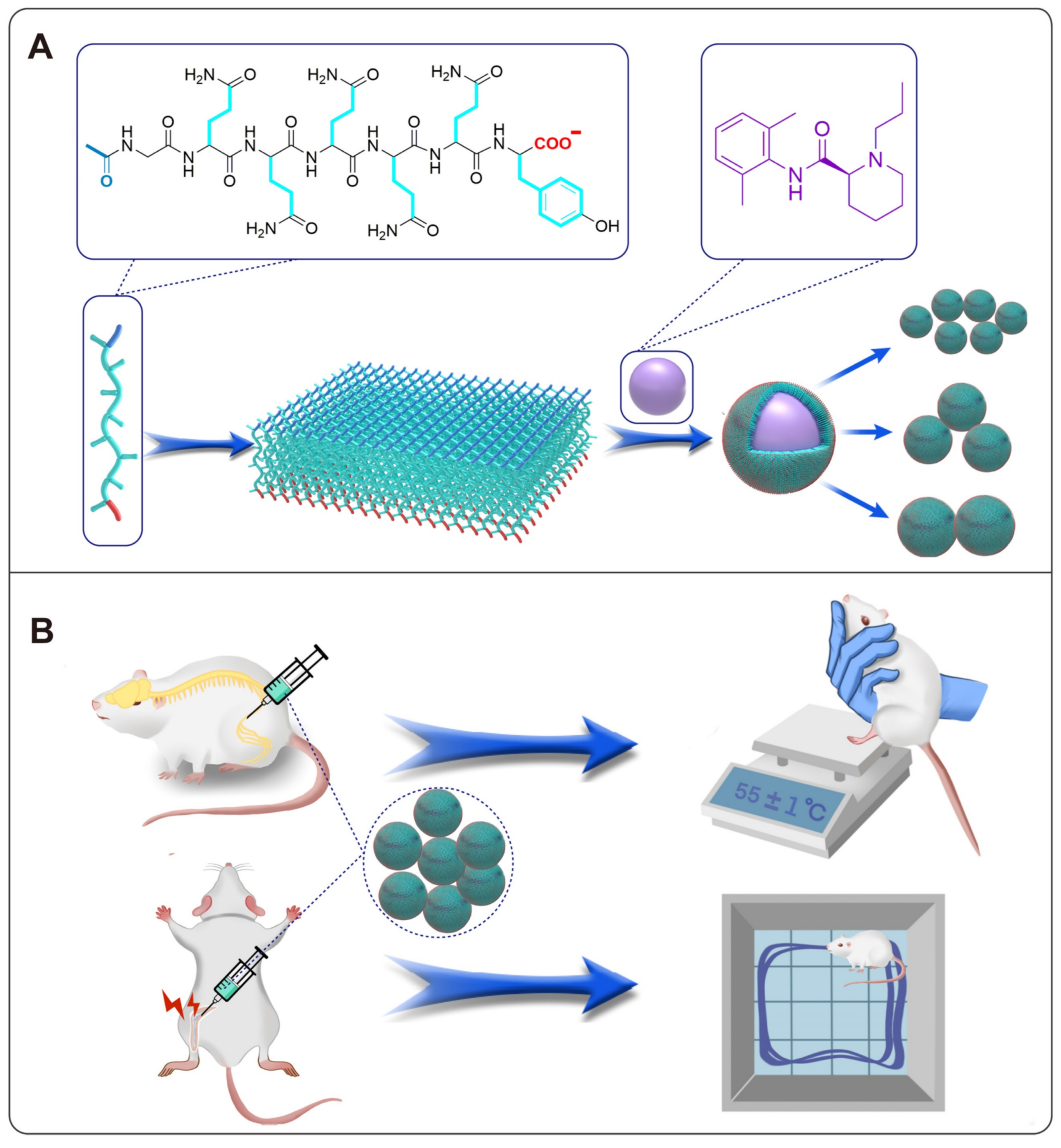
Illustration of self-assembling and drug-loading mechanisms of AG peptide (A), and the evaluation of their long-acting analgesic efficacy in animal models (B).

Following these ideas, we proposed a potential strategy for preparing LA base particles and optimizing their particle size to pursue long-acting formulations. Briefly, we designed a SAP with the sequence of Ac-GQQQQQY (abbreviated as AG), which could self-assemble into flexile nanosheets with negative charge and encapsulate ropivacaine base (RB) in a top-down manner. The size of RB particles could be adjusted by controlling the ratio between the peptide and RB, and an optimal size of RB particles was determined by a serial of studies including release behavior *in vitro*, pharmacokinetics and pharmacodynamics in rat sciatic nerve block model, as well as related toxicity evaluation. Based on the optimized size, the effective duration and safety of RB particles at various concentrations were evaluated. Finally, long-acting analgesia of the formulation was evaluated in a rat total knee replacement model (Figure 1).

## Methods

### Materials

Lyophilized AG peptide powder (purity > 98%) was purchased from GenScript Biotech (Nanjing, China). RB with a purity of 98% and RH with a purity of 99% were purchased from Macklin Biochemical Technology Co., Ltd (Shanghai, China). Pyrene (Sigma-Aldrich Co., USA), thioflavin-T (ThT, Aladdin Biochemical Technology Co., Ltd., Shanghai, China), and 1.33% bupivacaine liposomes injection (BUP@LS, Hengrui Pharmaceuticals Co., Ltd, Jiangsu, China) were used in this study.

### Formulation preparation

Lyophilized AG peptide powder was dissolved in phosphate buffer (PB) to get AG solution with a final concentration of 5 mM, and pH of the solution was adjusted to 5, 7 or 9 as needed. To encapsulate RB with AG peptide, AG solution (pH 7) was mixed with RB powder and stirred magnetically for 4-6 h to get a milky suspension, which was further treated in an ultrasound bath for 15-20 min. The size of RB@AG particles was adjusted by changing the theoretical mass concentration of RB in AG solution at a fixed concentration of 5 mM. Specifically, 1% RB in AG was used to prepare small nanoparticles (RB@AG-S), 6% RB in AG was used to prepare medium microparticles (RB@AG-M), and 24% RB in AG was used to prepare large microparticles (RB@AG-L). Different particle suspensions were directly used for characterization experiments, or particles were collected by centrifuge and resuspended with PBS to required concentration for release and animal studies. Pyrene@AG microparticles with the final pyrene concentration of 0.5% was prepared in a similar way. All formulations prepared in this study were stored at 4 °C before use.

### Characterization of peptide and drug-loading particles

To analyze the secondary structure of AG, circular dichroism (CD) was used to measure its CD spectrum at different pH. The peptide's self-assembling ability was investigated by a thioflavin T (ThT) binding fluorescence test, based on which its critical aggregation concentration (CAC) at different pH was determined. To characterize non-covalent interaction in the self-assembling structure of AG at different pH, pyrene fluorescence was measured to analyze hydrophobic interaction, while Fourier transform infrared spectroscopy (FTIR) was used to analyze hydrogen bond interaction. For morphological study, dynamic light scattering (DLS) was used to character the size and zeta potential, while transmission electron microscopy (TEM) and atomic force microscopy (AFM) were used to observe the self-assembling structure of AG at different pH.

For characterization of the peptide-encapsulated RB particles, scanning electron microscopy (SEM) and fluorescence microscopy were used to observe their detailed structures. DLS was used to analyze their particle size, surface area and zeta potential. FTIR and X-ray photoelectron spectroscopy (XPS) were used to analyze their chemical compositions. Powder X-ray diffraction (XRD) was used to analyze their crystal structure. Pyrene fluorescence was used to analyze the existence of monomers and excimers in the pyrene@AG particles. Detailed procedures of these characterization methods were described in the Supplementary Materials.

### Drug-loading capacity (DC) and encapsulation efficiency (EE)

Prepared RB@AG formulation was filtered using a 0.22 μm filter to get the supernatant. Concentration of ropivacaine in the supernatant was detected using high performance liquid chromatography (HPLC, Shimadzu, Japan). DC and EE were calculated according to the following formulas:



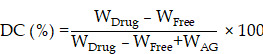





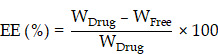



W_Drug_, total weight of drug; W_Free_, weight of free drug in the supernatant; W_AG_, weight of AG.

### Ropivacaine release test* in vitro*

Dialysis method was used to analyze the release behavior of different ropivacaine formulations. Briefly, 1 mL of each formulation was pipetted into a dialysis tube with the molecular weight cutoff of 100 KD (Float-A-Lyzer G2, SpectrumLabs Inc., USA). The dialysis tube was immersed into a 50 mL centrifuge tube filled with 40 mL of phosphate buffered saline (PBS, pH 7.4), which was constantly shaken at 80 rpm, 37 °C. At pre-set time intervals, a proper volume of dialysis buffer was extracted and fresh PBS of the equal volume was added into the centrifuge tube. The concentration of released ropivacaine at different time point was measured by HPLC as previously described 23, and the cumulative drug release rate was calculated accordingly.

### Animals

Male Sprague Dawley (SD) rats weighing 250-300 g (Dossy Biological Technology Co. Ltd; Chengdu, China) were housed in the Animal Experimental Centre of Sichuan University at 25 ± 1 °C, 60% humidity and a 12-hour light/dark cycle with *ad libitum* access to water and food. Prior to the experiment, the animals were acclimatized for 7 days to adapt to the experimental environment. In accordance with the principle of least harm and the results of preliminary experiment, the sample size was set as 6-8 per group. All animal experiment procedures were approved by the Animal Ethical Committee of West China Hospital, Sichuan University (Ethical approval number, 2020018A) and were performed in accordance with the criteria of the Animal Research: Reporting of In Vivo Experiments (ARRIVE) guidelines 31.

### Sciatic nerve block (SNB) model

To explore the effect of particle size on anesthetic efficacy with a modified SNB model 32, rats were randomly divided into 6 groups including normal saline (NS), AG, 1% RH, 1% RB@AG-S, 1% RB@AG-M and 1% RB@AG-L (n = 8). To evaluate the efficacy of formulations with different concentrations, rats were randomly divided into 5 groups including 2% RH, 4% RH, 2% RB@AG-M, 4% RB@AG-M and 6% RB@AG-M (n = 8). At different time point after drug injection, the modified hotplate test and the postural extensor thrust (PET) test were carried out to evaluate the sensory block and motor block, respectively. The detailed procedure of the SNB model was described in the Supplementary Materials.

### Pharmacokinetic study

To analyze drug release profile of RB@AG particles with different size *in vivo*, rats were randomly divided into 4 groups including 1% RH, 1% RB@AG-S, 1% RB@AG-M and 1 %RB@AG-L (n = 6). To evaluate that of RB@AG-M at different concentration, rats were randomly divided into 5 groups including 2% RH, 4% RH, 2% RB@AG-M, 4% RB@AG-M and 6% RB@AG-M (n = 6). After peri-sciatic injection of different formulations, the plasma ropivacaine concentration at preset time points were measured using liquid chromatography-mass spectrometry. The corresponding pharmacokinetic parameters were calculated using the Drug and Statistics (DAS, version 3.3.0) noncompartmental model for analysis. Detailed procedure of pharmacokinetic study was described in the Supplementary Materials.

### Cardiovascular and respiratory function measurement

The rat was kept in a steel holder with its tail exposed outside. The tail artery was cannulated with an intravenous catheter (22 G) and connected to a pressure sessor for blood pressure (BP) monitoring. A three-lead electrocardiogram (ECG) was monitored by placing electrodes subcutaneously in the left upper limb, right upper limb, and left lower limb. Thoracic movement was measured by a tension sensor for respiratory rate (RR). The three important vitals including BP, heart rate (HR) and RR was recorded by the BL-420 Biological Signal Acquisition and Analysis System (Techman, Chengdu, China). The baseline within 10 min before drug administration and vitals after peri-sciatic injection of different formulations were recorded.

### Total knee arthroplasty (TKA) model

SD rats were randomly divided into 5 groups including AG, 1% RH, 1.33% BUP@LS, 6% RB@AG-M and normal group (n = 8). Except for the normal group, all rats in other groups received TKA surgery as previously described 33, 34. The locomotor activities of rats at 6, 24 and 48 h after TKA were evaluated through open field test, which reflected the postoperative pain indirectly. Detailed procedure of the TKA model was described in the Supplementary Materials.

### Tissue harvesting and histological study

In the SNB model, rats were euthanized by cervical dislocation on day 4 and 14 after drug injection to harvest sciatic nerve and adjacent muscle. In the TKA model, rats were euthanized on day 14 after drug injection to harvest knee joint (1 cm length) of the surgery side. Rats used for cardiovascular and respiratory function study were euthanized on day 14 after drug injection to harvest important organs including heart, liver, spleen, lung and kidney. Harvested tissue and organs were fixed in 10% formaldehyde, embedded in paraffin and sectioned for staining with haematoxylin and eosin (HE) or toluidine blue. All stained sections were observed under optical microscope. The sections of sciatic nerve and muscle were evaluated for the inflammation (0-4), myotoxicity (0-6) 35, 36 and axonal degeneration (0-4) 37. The sections of different organs and knee joints were evaluated for the inflammatory reaction and structural changes.

### Stability evaluation of RB@AG formulation

Freshly prepared 6% RB@AG-M formulation was stored at 4 °C for eight weeks to evaluate its stability. DLS and SEM were used to monitor the change of particle size, surface charge and morphology. Free ropivacaine concentration and EE were measured at different time monitor the change composition. SNB model as described above was used to evaluate the anesthetic efficacy of the formulation at the end of the stability experiment.

### Statistical analysis

SPSS software (version 27, IBM Corp. USA) was used to analyze all data. Quantitative data were tested for normality using the Shapiro-Wilk test. Variables with normal distribution were described as mean ± standard deviation (SD), and differences between groups were compared using unpaired Student's t-test for two groups, and one-way ANOVA for more than two groups, followed by LSD or Dunnett T3 post-hoc test. Otherwise, variables non-normally distributed were presented as median (25th and 75th percentiles) and compared by Manne-Whitney test for two groups, while nonparametric tests for more than two groups followed by Bonferroni post-hoc test. Detailed statistical methods for different experiments were shown in corresponding figure legends or tables. All *p* values were bilateral and *p* < 0.05 was considered significant.

## Results

### AG self-assembled into nanosheets

**Figure 2:**
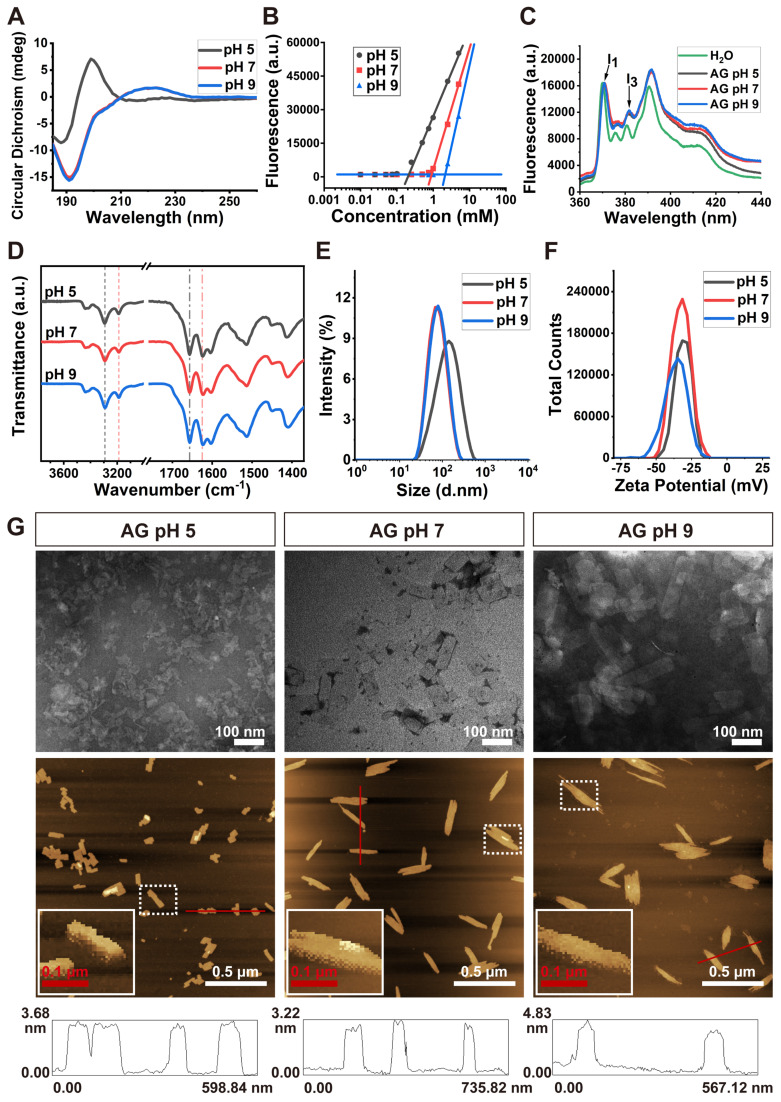
Self-assembly of AG at different pH. (A) CD spectra showing the secondary structure. (B) CAC value revealed by ThT-binding fluorescence. (C) Fluorescence spectra of pyrene in H_2_O or AG at different pH. (D) FTIR spectra. Dash lines indicate -NH_2_ peaks around 3300 cm^-1^ (black) and 3200 cm^-1^ (red), respectively, and dash-dot lines indicate -CONH- peaks around 1660 cm^-1^ (black) and 1625 cm^-1^ (red), respectively. (E) Size distribution. (F) Zeta potential. (G) Morphology of nanosheets formed by AG at different pH. Top panel, TEM images; middle panel, AFM images, with enlarged squares showing the detailed 3D images of nanosheets; bottom panel, line profile analysis indicating the height of nanosheets.

As shown in Figure 2A, CD spectra indicated that the AG peptide took *β*-sheet conformation only at pH 5, while at pH 7 or 9 it took irregular secondary structure, which has been believed to be unfavorable for self-assembly. Surprisingly, as indicated by the increase of ThT-binding fluorescent intensity at 495 nm, the peptide could self-assemble at various pH. As shown in Figure 2B and Figure S2, although AG at pH 5 showed stronger self-assembling ability with a lower CAC value, at pH 7 and 9 the peptide could also self-assemble with higher CAC values.

As shown in Figure 2C, an increase of I3 peak was observed when pyrene was mixed with AG at various pH, which indicated that the self-assembly of AG was driven by hydrophobic interaction. On the other hand, in the FTIR spectrum, part of the -CONH- peaks shifted from 1660 cm^-1^ to 1625 cm^-1^, while part of the -NH_2_ peaks shifted from 3300 cm^-1^ to 3200 cm^-1^ (Figure 2D). These FTIR peaks indicated that only a part of the peptide's -CONH- and -NH_2_ groups were involved in intermolecular hydrogen bond, suggesting its relatively loose self-assembling behavior.

DLS analysis showed that the size of AG at pH of 5, 7 and 9 was (121.5 ± 6.5), (71.4 ± 1.4) and (69.1 ± 2.8) nm, respectively (Figure 2E), with zeta potential of (-30.6 ± 0.9), (-34.2 ± 1.0) and (-37.2 ± 2.0) mV, respectively (Figure 2F). Obviously the strong negatively charge came from the dissociation of terminal carboxyl group on the peptide which slightly changed with pH change. At pH of 5 the surface charge was relatively weaker, which might cause the aggregation of self-assembling structures and lead to bigger size distribution revealed by DLS. However, at pH around 7 or above, strong negative charge might endow the self-assembling and drug loading system with high dispersity. TEM and AFM images revealed that AG self-assembled into nanosheets at different pH (Figure 2G). The height of the nanosheets formed by AG at pH of 5, 7 and 9 was (3.10 ± 0.11), (3.15 ± 0.11) and (3.60 ± 0.13) nm, respectively, which were close to the length of a peptide monomer (2.86 nm as estimated in Figure S3).

These morphological results confirmed the self-assembling mechanism proposed in Figure 1, i.e. the peptides were driven by the hydrophobic interaction among their sidechains and aligned side by side to form monolayer nanosheets. The subtle increase in the height of the nanosheets might reflect the slight change of the length of the peptide monomer when the peptide's secondary conformation transformed from compact *β*-sheet to freely extended irregular secondary structure. All these results indicated that self-assembly of AG was relatively stable over a wide range of pHs, but the structure was looser and more flexible at neutral and slightly alkalic pH, which provided a basis for encapsulating hydrophobic molecules in a soft-coating manner.

### Encapsulation of hydrophobic RB with AG nanosheets

**Figure 3:**
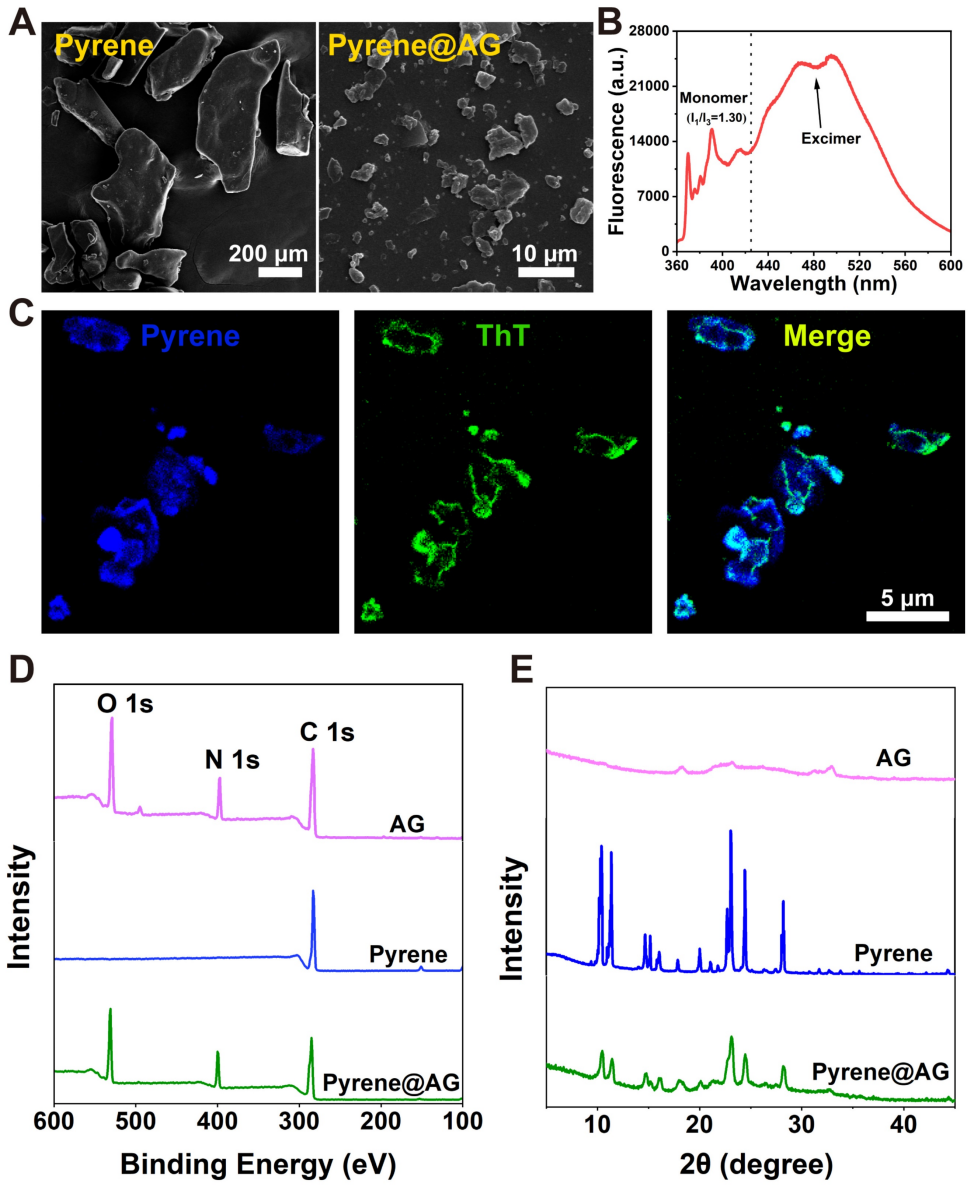
Characterization of pyrene@AG particles. (A) SEM images of pyrene and pyrene@AG particles. (B) Fluorescence spectrum of pyrene@AG particles. (C) Fluorescent images of pyrene@AG particles stained with ThT. (D) XPS spectra. (E) XRD spectra.

Firstly, pyrene was used as a model to prove the capacity of AG nanosheets to encapsulate hydrophobic drugs. As a highly hydrophobic molecule, pyrene could not dissolve or disperse in water, and SEM revealed that the drug formed insoluble crystals with size of hundreds of micrometers. On the contrary, pyrene could be well dispersed in AG and formed a milky suspension, which contained irregular pyrene@AG microparticles with size of less than 10 μm (Figure 3A and Figure S4).

As shown in Figure 3B, pyrene@AG suspension showed both monomer peaks between 360-440 nm and much stronger excimer peaks around 480 nm, which indicated that pyrene in the suspension mainly existed as microparticles. As shown in Figure 3C, fluorescence microscope images confirmed that AG nanosheets formed a layer of flexible soft-coating around the surface of irregular pyrene microparticles, providing an efficient encapsulation in a top-down manner. XPS analysis revealed the existence of oxygen and nitrogen on the surface of pyrene@AG particles, which further proved the existence of AG layer coating around the pyrene particles (Figure 3D). On the other hand, XRD patterns indicated that the crystal structure of pyrene@AG microparticles were slightly different from that of unencapsulated pyrene particles (Figure 3E).

**Figure 4:**
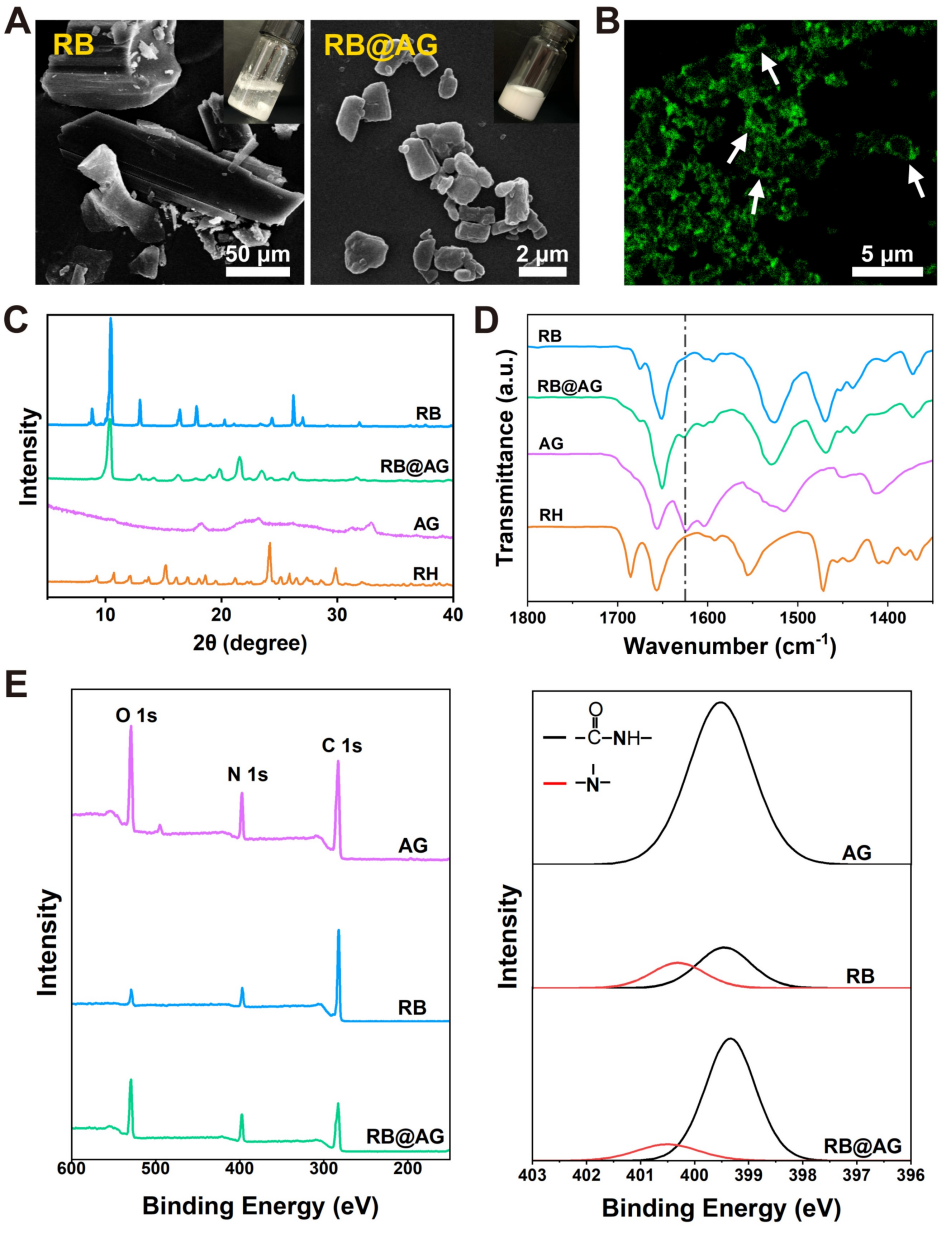
Characterization of RB@AG particles. (A) SEM images of RB and RB@AG particles. Pictures in the insertions show the appearance of the suspensions. (B) Fluorescent image of RB@AG particles stained with ThT. XRD spectra (C) and FTIR spectra (D) of RB, RB@AG, AG and RH. Dash-dot line indicates the -CONH- peak at 1625 cm^-1^. (E) XPS spectra of AG, RB and RB@AG. Left: original XPS survey spectra; Right: high-resolution N 1s spectra deconvolution analysis.

Similar top-down approach was used to prepare RB particles encapsulated with AG nanosheet (RB@AG). As shown in Figure 4A, the size of insoluble RB particles were tens to hundreds of micrometers, while they could be broken into much smaller microparticles and well-dispersed in AG, forming a milky suspension. As shown in Figure 4B, ThT-staining image of RB@AG microparticles clearly exhibited the existence of AG nanosheets coating around the RB microparticles.

As shown in Figure 4C, the XRD pattern of RB@AG was quite different from that of RH, and there were a few feature peaks identical with that of RB, which suggested that in the formulation the drug existed as RB crystals with the crystal structure slightly modified by the peptide coating. As shown in Figure 4D, FTIR spectrum of RB@AG was quite different from that of RH but was similar to that of RB, which further confirmed that RB kept their base form in the formulation rather than dissolved as RH. The downward peak at 1625 cm^-1^ also appeared in the spectrum of RB@AG, indicating the co-existence of RB and self-assembled AG in the formulation. Similarly, both the original XPS survey spectra and the high-resolution N 1s spectra deconvolution analysis also clearly showed the co-existence of RB and AG peptide in the micro-particle formulation (Figure 4E).

### Effect of particle size on release rate and effective duration

**Figure 5:**
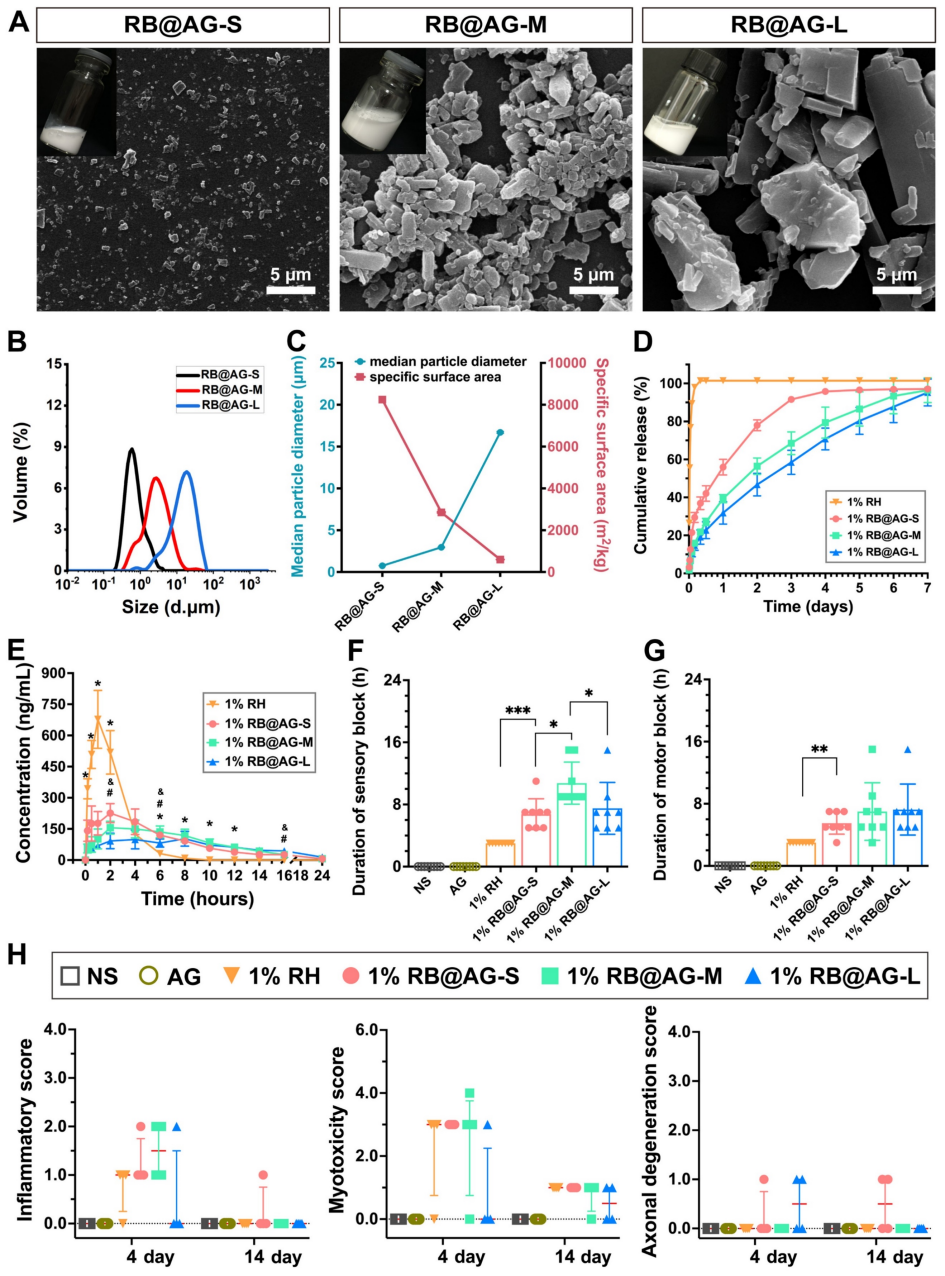
Characterization, release profile and pharmacodynamic study of RB@AG particles with different size. (A) SEM images. (B) Size distribution plots. (C) The change of surface area with particle size. (D) Drug release profiles *in vitro* of 1% RH and 1% RB@AG particles with different size (n = 3)*.* (E) Change of plasma ropivacaine concentration of 1% RH and 1% RB@AG particles with different size (n = 6). *, *p* < 0.05, three 1% RB@AG formulation groups compared with 1% RH, respectively; # and &, *p* < 0.05, 1% RB@AG-S and RB@AG-M group compared with RB@AG-L group, respectively; one-way ANOVA followed by LSD or Dunnett T3 post-hoc test. Duration of sensory block (F) and motor block (G) of different formulations in rat SNB model (n = 8). *, *p* < 0.05; **, *p* < 0.01; ***, *p* < 0.001. (H) Inflammatory score, myotoxicity score, and axonal degeneration score were evaluated 4 or 14 days after drug injection in the SNB model. Data were shown as mean (SD) for F and G, or median (25th and 75th percentiles) for H, and compared using nonparametric tests followed by Bonferroni post-hoc test.

As shown in Figure 5A, SEM images revealed different RB@AG nano/micro-particles obtained by changing the concentration of RB in the preparation process. As analyzed by DLS, averaged size of different particles was 0.75 ± 0.01 μm for RB@AG-S, 2.96 ± 0.04 μm for RB@AG-M and 16.70 ± 0.10 μm for RB@AG-L, respectively (Figure 5B and 5C). The specific surface area was (8247.67 ± 21.08) m^2^/kg for RB@AG-S, (2855.67 ± 29.57) m^2^/kg for RB@AG-M and (597.17 ± 2.65) m^2^/kg for RB@AG-L respectively, which clearly showed that the specific surface area drastically decreased with increasing particle size (Figure 5C). Furthermore, zeta potential of each formulation was (-35.2 ± 0.2) mV for RB@AG-S, (-37.2 ± 0.9) mV for RB@AG-M and (-31.9 ± 0.6) mV for RB@AG-L respectively, which suggested that in all formulations the RB particles were well-encapsulated by negatively charged AG nanosheets (Figure S5). Based on this top-down strategy in which drug particles were encapsulated by soft AG nanosheets, very high DC and EE could be achieved. For RB@AG-S, RB@AG-M and RB@AG-L, the DC and EE were 64.8% and 84.6%, 92.7% and 97.2%, 98.1% and 99.6%, respectively.

In the drug release study *in vitro*, 1% RH released 55.65% of its total drug within half an hour and almost achieved 100% release within 4 h. On the contrary, all the RB@AG formulations released slowly and did not show such a burst release. RB@AG-S, RB@AG-M and RB@AG-L formulations respectively released 29.44%, 15.90% and 14.74% of the drug within the first 4 h, and they respectively needed 3 days, 6 days, and 7 days to reach a release rate over 90% (Figure 5D). It was obvious that the release rate of different formulations was determined by their particle size, with smaller particles showing a faster release rate.

The changes of plasma ropivacaine concentration in the pharmacokinetic study were shown in Figure 5E. The time to maximum plasma concentration (T_max_) of 1% RH was (1.17 ± 0.41) h, while that of all 1% RB@AG formulations were longer than 2 h. Similarly, the mean retention time (MRT) of 1% RH was also shorter than that of each RB@AG formulation, which indicated a sustained drug release by the RB@AG formulations *in vivo*. Furthermore, the maximum blood concentration (C_max_) of 1% RH was (687 ± 129) μg/L, while that of RB@AG-S, RB@AG-M and RB@AG-L was (241 ± 53), (175 ± 25) and (119 ± 43) μg/L, respectively. The much lower C_max_ than that of 1% RH suggested that the risk of systemic toxicity of RB@AG formulations was much lower than RH at the same dose. All these data confirmed that the release rate of RB@AG formulations was slowed down as the particle size increased. Furthermore, it should also be noted that the area under the curve (AUC) of different RB@AG formulations slightly reduced with the increase of particle size, and the RB@AG-L particle with the lowest AUC may not fully released its drug content. The detailed pharmacokinetic results and corresponding analysis were shown in Table S1.

In the rat SNB model, 1% RH, 1% RB@AG-S and 1% RB@AG-M reached the MPE of 100% and the PET below 20 g within 10 min after injection, indicating their rapid onset, while 1% RB@AG-L achieved 100% MPE and PET below 20 g within 30 min, suggesting its relatively slower onset, which was in accordance with their release behavior* in vitro* (Figure S6). As shown in Figure 5F, 1% RH showed a sensory block duration of 3 h, while for the slow-releasing RB@AG formulations, sensory block duration was (6.75 ± 1.98) h for 1% RB@AG-S, (10.75 ± 2.71) h for 1% RB@AG-M and (7.50 ± 3.34) h for 1% RB@AG-L, respectively. Similarly, duration of motor block was 3 h for 1% RH, (5.50 ± 1.41) h for 1% RB@AG-S, (7.00 ± 3.70) h for 1% RB@AG-M and (7.25 ± 3.28) h for 1% RB@AG-L, respectively (Figure 5G). The durations of nerve block of all RB@AG formulations were much longer than that of RH at the same concentration. Furthermore, they were even longer than 1.33% BUP@LS, a commercialized long-acting LA formulation (Figure S7). Since AG did not generate any anesthetic effect like NS while the dose of ropivacaine in all formulations were identical, it was obvious that the longer anesthetic duration was achieved by slow release of ropivacaine from RB@AG formulations. On the other hand, the sensory block duration of RB@AG-M was much longer than that of RB@AG-S and RB@AG-L, indicating that the formulation consisting in medium scaled particles could generate a more appropriate release rate and was more efficient in prolonging the duration of the drug.

Similar to NS, the AG peptide did not show any sign of local toxicity including inflammation, myotoxicity and neurotoxicity (Figure 5H and S8). As shown in Figure S9, the peptide did not show obvious toxicity on cultured muscle and nerve cells either, suggesting its excellent biocompatibility and safety. Although all formulations containing 1% ropivacaine caused mild to moderate local toxicity on day 4 post-injection, there was no significant difference between the nano/micro-particle groups and the free RH group. Furthermore, in all groups the toxicity score decreased to a very low level on day 14 post-injection (Figure 5H and Figure S8). These results indicated that the mild toxicity observed for our formulations was caused by the inevitable toxicity of 1% ropivacaine, which is clinically acceptable and could recover over time.

All these results suggested that by virtue of their slow-release profile, the RB@AG formulations had the advantage of longer nerve block duration compared with RH at the same concentration, without causing extra local damage. In addition, RB@AG with smaller particles released much faster both *in vitro* and *in vivo*, while particles with diameter large than 10 μm released too slow and inefficiently, leading to less reliable efficacy. In summary, RB@AG-M mainly consisting of micro-particles with the average size of 2.96 ± 0.04 μm could achieve an optimal release rate and produce a longer anesthetic effect with considerable safety.

### Different anesthetic duration achieved by RB@AG-M formulations

**Figure 6:**
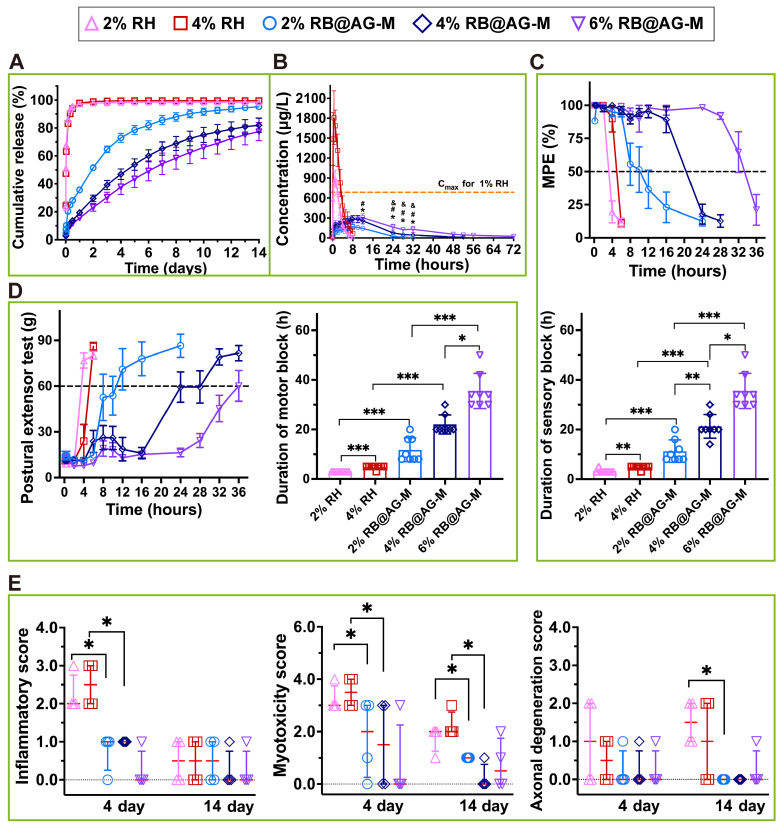
Release profile, anesthetic efficacy, and local toxicity evaluation of RB@AG-M and RH formulations at different concentration. (A) Release profile *in vitro* (n = 3). (B) Change of plasma ropivacaine concentration of different formulations (n = 6). The dotted line shows the maximum plasma concentration of 1% RH. *, *p* < 0.05, 2% RB@AG-M versus 4% RB@AG-M; #, *p* < 0.05, 2% RB@AG-M versus 6% RB@AG-M; &, *p* < 0.05, 4% RB@AG-M versus 6% RB@AG-M; one-way ANOVA followed by LSD or Dunnett T3 post-hoc test. (C) MPE and duration of effective sensory block. The dotted line shows MPE value of 50%, values more than which are defined as effective sensory block (n = 8). (D) PET value and duration of effective motor block. The dotted line indicated PET value of 60 g, values above which are defined as motor block recovery (n = 8). (E) Inflammatory score, myotoxicity score and axonal degeneration score. Data were expressed as mean (SD) for C and D, or median (25th and 75th percentiles) for E, and compared using nonparametric tests followed by Bonferroni post-hoc test. *, *p* < 0.05; **, *p* < 0.01; ***, *p* < 0.001.

Then RB@AG-M was used to prepare formulations with ropivacaine concentration of 2%, 4% and 6% to evaluate their efficacy. Soluble RH formulations with concentration of 2% and 4% were used as control, while 6% RH was not included because RH could not completely dissolve in H_2_O at this concentration. As shown in Figure 6A, 2% and 4% RH formulations showed an identical burst release profile, with more than 90% of the drug released within the first 8 h. On the contrary, 2% RB@AG-M released only 24.8% of the drug within 8 h, and it needed 9 days to reach the release rate of 90%. Compared with 2% RB@AG-M, the 4% and 6% RB@AG-M formulations exhibited even slower release rates.

As shown in Figure 6B exhibiting the release profile* in vivo*, the T_max_ of 4% RH was (0.67 ± 0.26) h, which was close to that of 2% RH (0.83 ± 0.26) h, indicating that the release rate of RH at different concentration was a similar burst release. On the contrary, T_max_ values of RB@AG-M formulations were prolonged as the concentration of ropivacaine increased, which suggested slow release of RB@AG-M formulations *in vivo* (Table S2)*.* Furthermore, C_max_ of 2% and 4% RH was (898 ± 144) and (1812 ± 395) μg/L, respectively, exhibiting a rapid C_max_ increase with the increase of drug concentration, which might increase the risk of systemic toxicity. On the contrary, C_max_ of RB@AG-M formulations only slightly increased as the concentration of ropivacaine increased, and even the C_max_ of 6% RB@AG-M was much lower than that of 1% RH (*p* < 0.01), indicating the safety of these slow-releasing formulations.

In the rat SNB model, all formulations reached the MPE of 100% and the PET below 20 g within 10 min after injection, which indicated that the RB@AG-M formulations caused effective nerve block as fast as the RH formulations. On the other hand, the MPE and PET of 2% and 4% RH quickly went back to normal values within several hours, while those of the RB@AG-M formulations could maintain at the effective values for a much longer time (Figure 6C and 6D). As shown in the statistic results in Figure 6C, the sensory block duration of 2% RH and 4% RH was (3.25 ± 0.71) and (4.75 ± 0.71) h, respectively, while that of 2%, 4% and 6% RB@AG-M was (11.25 ± 4.53), (21.25 ± 4.77), and (35.50 ± 7.07) h, respectively. Similarly, the motor block duration of 2% RH and 4% RH was 3 and (4.75 ± 0.71) h, respectively, while that of 2%, 4% and 6% RB@AG-M was (11.75 ± 4.83), (22.00 ± 3.85) and (35.50 ± 7.07) h, respectively (Figure 6D). Basically, the nerve block duration of RB@AG-M formulations was much longer than that of RH at the same concentration. More importantly, different block duration could be intendedly achieved by controlling the concentration of RB in the RB@AG-M formulation. On the contrary, increasing the concentration of free RH showed little effect in prolonging the nerve block duration.

For all the groups injected with RB@AG-M formulation, no obvious drug residues were observed at the injection site (Figure S10). Although mild inflammatory cell infiltration was observed on day 4, the inflammatory score got decreased on day 14. Similarly, mild and recoverable myotoxicity and axonal degeneration were also observed after injection of different RB@AG formulations (Figure 6E). However, compared with 1% RH used in clinic, there was no significant difference in the inflammation, myotoxicity and axonal damage evaluated for three RB@AG formulations (Table S3). On the other hand, moderate to severe inflammation and myotoxicity were observed in 2% and 4% RH group on day 4, which slightly mitigated on day 14. In addition, rats injected with 2% or 4% RH showed mild axonal degeneration on day 4, which got even worse on day 14. Compared with 1% RH or the RB@AG formulations at the same concentration, the local inflammatory cell infiltration, myotoxicity, and nerve damage of 2% and 4% RH was more serious (Table S3), indicating that the local injury got worse as the concentration of soluble RH increased. As a comparison, the local damage of 1.33% BUP@LS was also more serious than that of 1% RH (Figure S7B and Table S4).

### Low systematic toxicity of the RB@AG-M formulation

**Figure 7:**
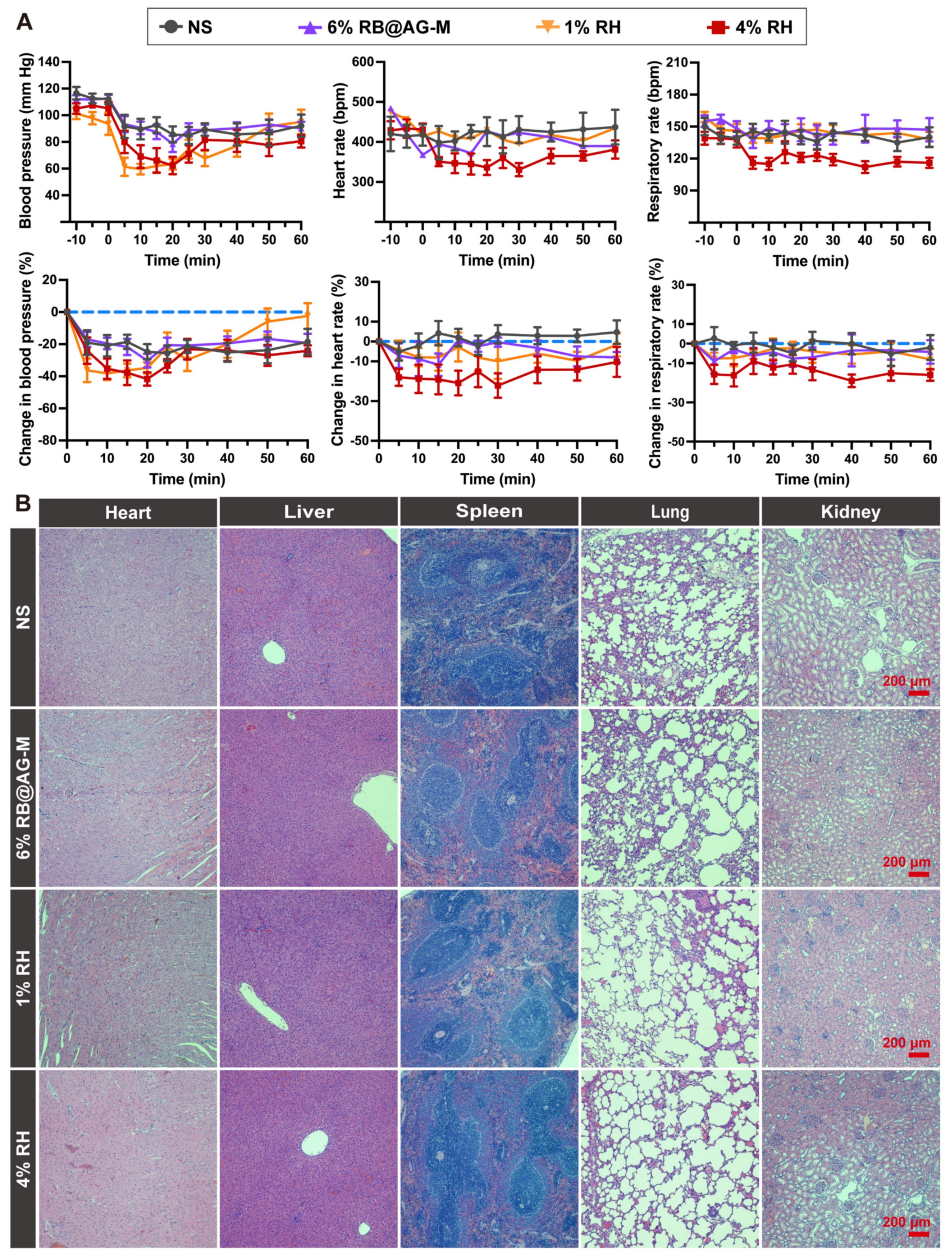
Systemic toxicity evaluation of RB@AG-M and RH formulation at different concentration. (A)The change in BP, HR and RR (n = 6). (B) HE staining of main organs after locally injected with ropivacaine formulations in SNB model.

To evaluate the systematic toxicity of the RB@AG-M formulation, we investigated the change in BP, HR and RR of rats injected with NS, 6% RB@AG-M, 1% RH or 4% RH. As shown in Figure 7A, although there was an obvious decrease of BP in all groups including the NS group, this might be attribute to the general anesthesia procedure needed for injection. However, the level of BP decrease caused by 6% RB@AG-M was close to that of the NS group, which was much better than the 1% RH and 4% RH groups. Furthermore, for the change of HR and RR, 6% RB@AG-M showed effect similar to NS and 1% RH, while 4% RH exhibited more significant impact on HR and RR. These results were also coincident with the plasma ropivacaine concentration shown in Figure 6B, where the C_max_ of 6% RB@AG-M was well-controlled below the C_max_ of 1% RH, while the C_max_ of 4% RH was much higher.

To evaluate the long-term toxicity of the RB@AG-M formulation, important organs including heart, liver, spleen, lung, and kidney of rats were harvested 14 days after local injection of different formulations. As shown in Figure 7B, HE staining images revealed no obvious damage on any organ in any of the four groups, suggesting that neither ropivacaine nor the peptide material would cause long-term toxicity on these important organs.

### Analgesic effect of RB@AG-M formulation in TKA model

**Figure 8:**
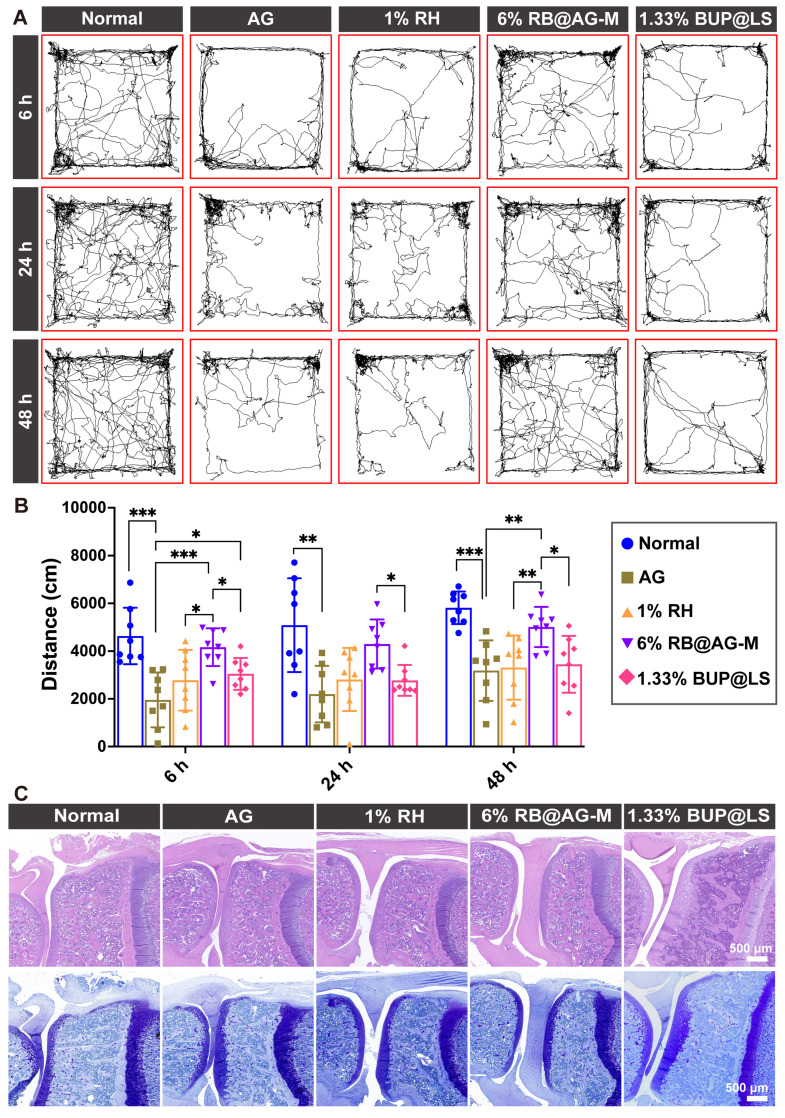
Long-acting analgesic efficacy of 6% RB@AG-M in rat TKA model. (A) Representative images of trace of rats in open field test. (B) Total distance of rats in the open field (n = 8). Data was shown as mean (SD) and compared using one-way ANOVA followed by LSD post-hoc test. *, *p* < 0.05; **, *p* < 0.01; ***, *p* < 0.001. ((C) Representative microscopy images of HE (upper panel) and toluidine blue (lower panel) stained sections of knees from different groups.

The long-acting analgesia effect of 6% RB@AG-M was further evaluated in a rat TKA surgery model, which is a conventional postoperative pain model mimicking the clinical TKA surgery. The travel distance of rats in the open filed after TKA surgery was used to assess postoperative pain (Figure 8A and 8B). Compared with normal rats, activities of rats in the peptide group reduced significantly, suggesting that the surgery caused postoperative pain and the peptide as carrier material did not show any analgesic effect. Clinically used 1% RH and 1.33% BUP@LS as the positive control groups only mildly improved activity of rats at 6 h post-surgery, suggesting their very short duration of analgesic effect. However, the average total distance of rats injected with 6% RB@AG-M was much longer than that of rats in the 1% RH group and 1.33% BUP@LS group at 6 h, 24 h and 48 h after surgery, which indicated that 6% RB@AG-M could efficiently inhibit postoperative pain induced by TKA surgery within 48 h. On the other hand, no arthritic damage was observed for animals in all groups, suggesting the excellent safety of this formulation (Figure 8C).

### Stability of the RB@AG-M formulation

**Figure 9:**
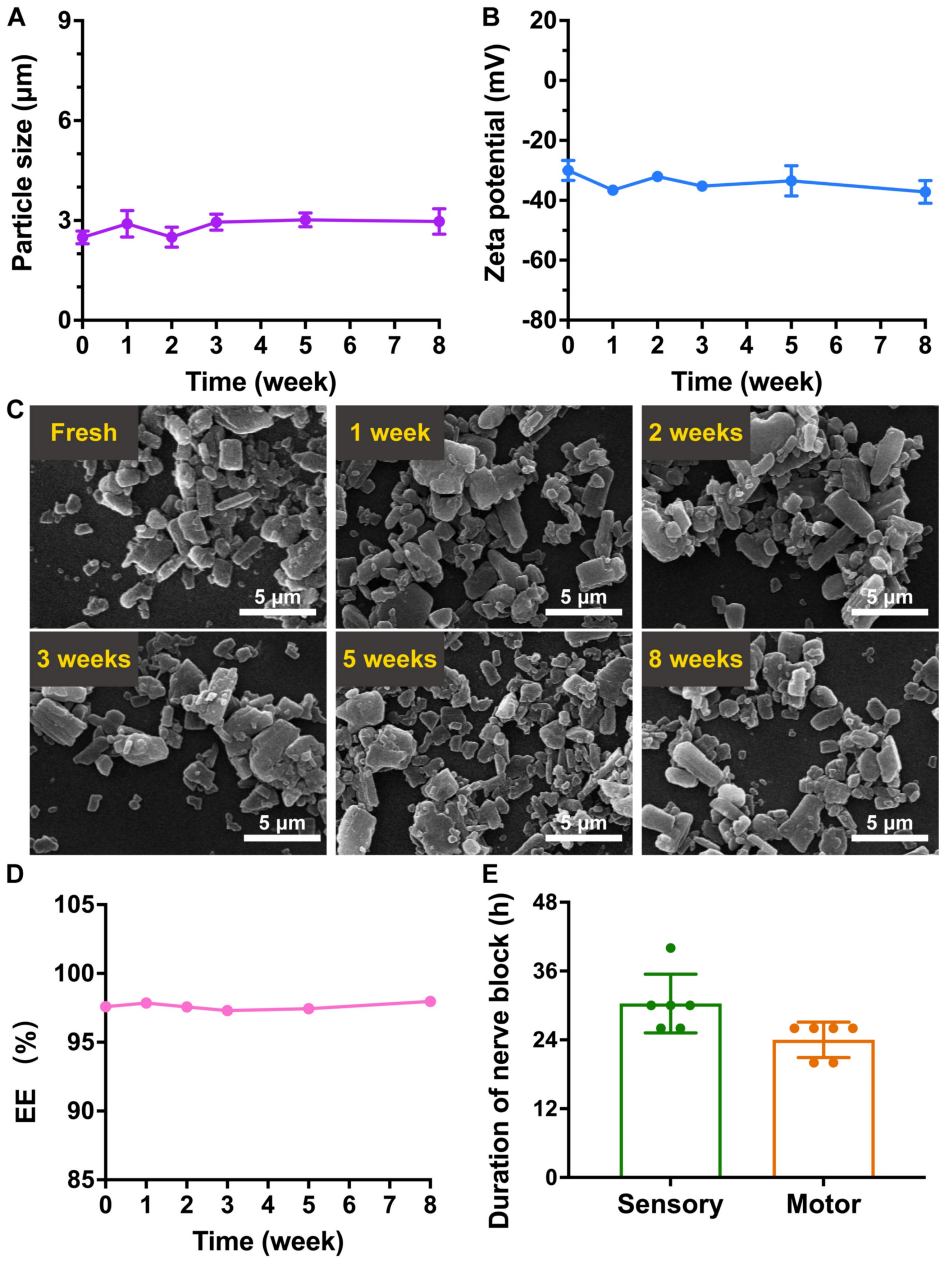
The stability of RB@AG-M formulation. The change of size (A), zeta potential (B) and morphology revealed by SEM (C). (D) The change of EE of 6% RB@AG-M formulation after stored for different time. (E) The nerve block duration of 6% RB@AG-M formulation after stored for eight weeks (n = 6).

The RB@AG-M formulation was stored at 4 °C for eight weeks to evaluate its stability. As revealed by DLS, average size of the particles was kept around 2.5~3.0 μm, and the zeta potential was kept around -30~-37 mV, suggesting the stability of particle size and surface charge (Figure 9A and 9B). As shown in Figure 9C, RB@AG-M kept its morphology of microparticles during the storage, with no obvious size or morphological change observed.

Furthermore, the EE of ropivacaine in the 6% RB@AG-M formulation was also monitored for eight weeks to confirm if there was any undesired drug leakage during the storage. As shown in Figure 9D and Table S5, the EE of the 6% RB@AG-M formulation kept around 97% for eight weeks, suggesting the stability of drug content in the formulation. Based on these results, anesthetic efficacy of the 6% RB@AG-M formulation after 8 weeks of storge was assessed in rat SNB model. As shown in Figure 9E, the sensory and motor block duration of the stored formulation was 30.33 ± 5.13 h and 24.00 ± 3.10 h, respectively, which were a little shorter than the fresh formulation. However, the sensory block duration of stored formulation had no significant difference with that of fresh formulation. All these results demonstrated that the RB@AG-M formulation was relatively stable after stored at 4 °C for eight weeks.

## Discussion

In the management of postoperative pain, LAs such as lidocaine, bupivacaine and ropivacaine have been conventionally used in their highly water-soluble hydrochloride forms to ensure the convenient preparation and injection of aqueous formulations. However, high water-solubility also leads to fast release, diffusion and clearance of LAs and limit their duration of effect. In our study, RH formulations exhibited burst release *in vitro* and *in vivo*, generating anesthetic and analgesic effects for only several hours (Figure 5 and 6). On the contrary, LAs in their base form are less soluble, providing ideal candidates for preparing slow-releasing formulations. Unfortunately, commercially available base-form LAs are usually very big crystals and cannot be evenly dispersed in water solution (Figure 4A), making them unsuitable for accurate injection to achieve a reliable anesthetic effect. Actually, in clinical practice big crystals usually exhibited inefficient effect.

Behind this dilemma is a quite simple principle which seems to be neglected so far: An ideal long-acting formulation need a proper, not just slow, release rate to keep local drug concentration within a range between the effective and toxic thresholds. To this end, controlling the particle size of drug crystals is a promising strategy to achieve a suitable release rate 26, 28. Following this idea, in this study we compared the release profile and anesthetic efficacy of RB particles with different size. As shown in Figure 5, RB particles with a medium size of 2.96 ± 0.04 μm exhibited the best anesthetic efficacy in terms of duration, variability, and safety. Unlike many previous studies simply fabricated LA particles with a certain size, this study for the first time pointed out the necessity and possibility to adjust the size distribution of nano/micro-particles when pursuing long-acting LA formulations. By confirming that different release rate could be achieved by LA particles with different size, our study established the basic mechanism for controlling the release profile of LA formulations in an even more deliberate manner. In future studies, regulating the ratio of particles with different size in a complex formulation might achieve an even better release profile for even longer analgesic duration.

For different types of surgery causing postoperative pain with different duration, it would be ideal to use LA formulations with different anesthetic duration, which could exactly cover the time window of pain without causing extra block of sensory or motor function. Compared with clinical hydrochloride formulations, the RB@AG-M particle developed in our study provided such a convenient choice by adjusting the drug concentration, which might be used to meet the requirement of different analgesic duration in different types of surgery (Figure 6). On the contrary, raising the concentration of RH solution not only failed to substantially prolong the anesthetic duration, but also it caused more serious side effects including local and systematic toxicity (Figure 6 and 7A). Furthermore, since the drug concentration of commercialized BUP@LS was only 1.33%, it was not surprising that in our study this formulation with the same injection volume generated effective nerve block for only 4-6 hours (Figure S7A), which was similar to a previous study 35. In clinical application, several studies also reported that the analgesic efficacy of Exparel^®^ was limited 38, 39, likely due to its relatively low drug concentration. In our study, the RB@AG-M formulation could be prepared at the concentration up to 6% and generate sustained analgesia for up to 48 h in a TKA model (Figure 8), providing a sufficient long and adjustable analgesic effect in treating different types of long-lasting postoperative pain.

In this study, the LA microparticle formulations were prepared by using SAP nanosheets to encapsulate RB particles in a soft-coating manner, which have shown two major advantages. On one hand, by forming a thin layer of peptide nanosheet coating around RB nano/micro-particles, this system achieved much higher capacity to encapsulate ropivacaine compared with previous strategies using traditional materials 40-42, which might greatly cut down the cost of fabrication. On the other hand, compared with our previously reported bottom-up strategies which were somehow complicated by relying on special interaction between LAs and the carrier materials, the current top-down strategy was much more straightforward and facile in obtaining particles with different size. It should be pointed out that in this laboratory investigation the encapsulation protocol was still inefficient in obtaining particles with relatively narrow size distribution. However, considering the simplicity of this top-down strategy and availability of peptide materials via conventional peptide synthesis, in the future industrial equipment such as high-pressure homogenizer could be employed to better control the size and homogeneity of the drug particles, and scale up the production for potential clinical application.

Basically, the advantages mentioned above were based on the designer AG peptide, which could undergo self-assembly based on irregular secondary structure, through moderate hydrophobic interaction and hydrogen bond (Figure 2). This is quite different from most conventional SAPs systems, where the peptides usually contained many typical hydrophobic amino acids, making them prone to take *β*-sheet conformation, generate strong intermolecular hydrogen bond and form rigid and compact nanostructures 43, 44. Alternatively, AG was mainly composed of glutamine, an atypical hydrophobic amino acid with moderate hydrophobicity in its sidechain, so that it was able to form relatively looser nanosheet with high flexibility, making it efficient in soft-coating drug particles with irregular shape. Furthermore, strong negative surface charge brought by the terminal carboxyl group of the peptide also helped to enhance the dispersity of drug particles (Figure 3 and Figure 4). On the other hand, the self-assembling and encapsulation processes were completely carried out in water without involving in any toxic organic solvents, resembling the important advantage of environment-friendly green synthesis processes 45, 46. As a peptide material composed of natural amino acids, its biocompatibility has also been well-confirmed in both cell and animal experiments (Figure S8 and S9). All these features have ensured that the soft-coating method based on AG peptide provides a safe, facile, and cost-effective strategy to obtain RB particles with different size.

Combining excellent biocompatibility of the peptide material and the well-controlled slow-release profile, the long-acting formulations developed in this study showed considerable safety in terms of local and systematic toxicity. As shown in Figure 6, even though the drug content in 6% RB@AG-M formulation was 6-times of that in the 1% RH formulation, the two formulations exhibited similar local toxicity, which was mild and could undergo self-recovery over time. This is very important for clinical application, since inevitable toxicity of LAs should be well controlled to ensure the recovery of normal sensory and motor function after the course of postoperative pain. On the other hand, due to the slow-release profile, the peak plasma drug concentration in the 6% RB@AG-M formulation was controlled even lower than the peak value of the 1% RH formulation which has been safely used in clinic. This is another important feature considering that except for blocking local sodium channels for anesthetic effect, ropivacaine absorbed into blood could also block other vital sodium channels in other organs, leading to life-threatening side effects. Due to the well-controlled plasma drug concentration, the 6% RB@AG-M formulation exhibited considerable systematic safety comparable to, or even better than the 1% RH formulation, indicating its potential safety in future clinical application.

Following safety and effectiveness, stability is also an important issue to be addressed for novel formulations on their way to clinical application. In a preliminary study, the 6% RB@AG-M formulation showed consistent size distribution, surface charge, morphology, drug content in particles and anesthetic efficacy when stored as suspension at 4 °C, suggesting its considerable stability (Figure 9). In further translational investigation, the formulation's stability under different storage condition and in even a longer period of time will be evaluated.

## Conclusions

In this study, we designed the AG peptide which could self-assemble into flexible nanosheets with negative charge. In a top-down manner, AG could efficiently encapsulate RB to form nano/micro-particles by forming soft-coating layers on the particle surface. Compared with free RH, RB@AG nano/micro-particles could slowly release ropivacaine both *in vitro* and *in vivo*, and the release rate decreased as the particle size increased, which consequently affected the anesthetic efficacy in SNB model. In the current study, RB@AG-M was chosen as the optimal formulation, which exhibited long-acting analgesic effects in rat SNB and TKA models. Considering the structural and chemical similarity of different LA molecules, this strategy could be readily applied on other clinically used LAs such as lidocaine and bupivacaine for exploring more long-acting formulations.

## Supplementary Material

Supplementary materials and methods, figures and tables.

## References

[1] The Lancet Public H (2022). Opioid overdose crisis: time for a radical rethink. Lancet Public Health.

[2] Humphreys K, Shover CL, Andrews CM, Bohnert ASB, Brandeau ML, Caulkins JP (2022). Responding to the opioid crisis in North America and beyond: recommendations of the Stanford-Lancet Commission. Lancet.

[3] Doleman B, Mathiesen O, Sutton AJ, Cooper NJ, Lund JN, Williams JP (2023). Non-opioid analgesics for the prevention of chronic postsurgical pain: a systematic review and network meta-analysis. Br J Anaesth.

[4] Joshi G, Gandhi K, Shah N, Gadsden J, Corman SL (2016). Peripheral nerve blocks in the management of postoperative pain: challenges and opportunities. J Clin Anesth.

[5] Liu T, Yang J, Wang Y, Jiang W, Luo Y, Feng X (2023). Interfascial plane block: a new anesthetic technique. Anesthesiol Perioper Sci.

[6] Svensson I, Sjöström B, Haljamäe H (2000). Assessment of pain experiences after elective surgery. J Pain Symptom Manage.

[7] Wang C, Yang J, Chang W (2022). PLGA-based microspheres containing ropivacaine and betamethasone for sciatic nerve block in mice. Pharm Dev Technol.

[8] Chung TW, Huang YY, Liu YZ (2001). Effects of the rate of solvent evaporation on the characteristics of drug loaded PLLA and PDLLA microspheres. Int J Pharm.

[9] Ratajczak-Enselme M, Estebe JP, Dollo G, Chevanne F, Bec D, Malinovsky JM (2009). Epidural, intrathecal and plasma pharmacokinetic study of epidural ropivacaine in PLGA-microspheres in sheep model. Eur J Pharm Biopharm.

[10] Epstein-Barash H, Shichor I, Kwon AH, Hall S, Lawlor MW, Langer R (2009). Prolonged duration local anesthesia with minimal toxicity. Proc Natl Acad Sci U S A.

[11] Richard BM, Ott LR, Haan D, Brubaker AN, Cole PI, Nelson KG (2011). The safety and tolerability evaluation of DepoFoam bupivacaine (bupivacaine extended-release liposome injection) administered by incision wound infiltration in rabbits and dogs. Expert Opin Investig Drugs.

[12] Foley PL, Ulery BD, Kan HM, Burks MV, Cui Z, Wu Q (2013). A chitosan thermogel for delivery of ropivacaine in regional musculoskeletal anesthesia. Biomaterials.

[13] Luu CH, Nguyen G, Le TT, Nguyen TN, Giang Phan VH, Murugesan M (2022). Graphene Oxide-Reinforced Alginate Hydrogel for Controlled Release of Local Anesthetics: Synthesis, Characterization, and Release Studies. Gels.

[14] Zhang Y, Shi K, Yang X, Chen W, Wang T, Kang Y (2023). Sustained release of levobupivacaine from temperature-sensitive injectable hydrogel for long-term local anesthesia in postoperative pain management. Biomaterials.

[15] Ji T, Li Y, Deng X, Rwei AY, Offen A, Hall S (2021). Delivery of local anaesthetics by a self-assembled supramolecular system mimicking their interactions with a sodium channel. Nat Biomed Eng.

[16] Zhang W, Ning C, Xu W, Hu H, Li M, Zhao G (2018). Precision-guided long-acting analgesia by Gel-immobilized bupivacaine-loaded microsphere. Theranostics.

[17] Yin QQ, Wu L, Gou ML, Qian ZY, Zhang WS, Liu J (2009). Long-lasting infiltration anaesthesia by lidocaine-loaded biodegradable nanoparticles in hydrogel in rats. Acta Anaesthesiol Scand.

[18] Karavasili C, Komnenou A, Katsamenis OL, Charalampidou G, Kofidou E, Andreadis D (2017). Self-Assembling Peptide Nanofiber Hydrogels for Controlled Ocular Delivery of Timolol Maleate. ACS Biomater Sci Eng.

[19] Michiue H, Kitamatsu M, Fukunaga A, Tsuboi N, Fujimura A, Matsushita H (2021). Self-assembling A6K peptide nanotubes as a mercaptoundecahydrododecaborate (BSH) delivery system for boron neutron capture therapy (BNCT). J Control Release.

[20] Liu J, Peng F, Kang Y, Gong D, Fan J, Zhang W (2021). High-Loading Self-Assembling Peptide Nanoparticles as a Lipid-Free Carrier for Hydrophobic General Anesthetics. Int J Nanomedicine.

[21] Peng F, Liu J, Zhang Y, Fan J, Gong D, He L (2022). Designer self-assembling peptide nanofibers induce biomineralization of lidocaine for slow-release and prolonged analgesia. Acta Biomater.

[22] Peng F, Liu J, Zhang Y, Zhao G, Gong D, He L (2022). Interaction Between Ropivacaine and a Self-Assembling Peptide: A Nanoformulation for Long-Acting Analgesia. Int J Nanomedicine.

[23] Peng F, Liu J, Chen J, Wu W, Zhang Y, Zhao G (2023). Nanocrystals Slow-Releasing Ropivacaine and Doxorubicin to Synergistically Suppress Tumor Recurrence and Relieve Postoperative Pain. ACS Nano.

[24] Hoerner E, Stundner O, Putz G, Steinfeldt T, Mathis S, Gasteiger L (2022). Crystallization of ropivacaine and bupivacaine when mixed with different adjuvants: a semiquantitative light microscopy analysis. Reg Anesth Pain Med.

[25] Hoerner E, Stundner O, Fiegl H, Gasteiger L (2023). Crystallization of short-acting and intermediate-acting local anesthetics when mixed with adjuvants: a semiquantitative light microscopy analysis. Reg Anesth Pain Med.

[26] Bragagni M, Beneitez C, Martin C, Hernan Perez de la Ossa D, Mura PA, Gil-Alegre ME (2013). Selection of PLA polymers for the development of injectable prilocaine controlled release microparticles: usefulness of thermal analysis. Int J Pharm.

[27] Kapate N, Clegg JR, Mitragotri S (2021). Non-spherical micro- and nanoparticles for drug delivery: Progress over 15 years. Adv Drug Deliv Rev.

[28] Ho MJ, Jeong MY, Jeong HT, Kim MS, Park HJ, Kim DY (2022). Effect of particle size on in vivo performances of long-acting injectable drug suspension. J Control Release.

[29] Huang H, Xue Q, Chen B, Xiong Y, Schneider J, Zhi C (2017). Top-Down Fabrication of Stable Methylammonium Lead Halide Perovskite Nanocrystals by Employing a Mixture of Ligands as Coordinating Solvents. Angew Chem Int Ed Engl.

[30] Pinna A, Lasio B, Piccinini M, Marmiroli B, Amenitsch H, Falcaro P (2013). Combining top-down and bottom-up routes for fabrication of mesoporous titania films containing ceria nanoparticles for free radical scavenging. ACS Appl Mater Interfaces.

[31] Percie du Sert N, Ahluwalia A, Alam S, Avey MT, Baker M, Browne WJ (2020). Reporting animal research: Explanation and elaboration for the ARRIVE guidelines 2.0. PLoS Biol.

[32] Lee KC, Wilder RT, Smith RL, Berde CB (1994). Thermal hyperalgesia accelerates and MK-801 prevents the development of tachyphylaxis to rat sciatic nerve blockade. Anesthesiology.

[33] Buvanendran A, Kroin JS, Kari MR, Tuman KJ (2008). A new knee surgery model in rats to evaluate functional measures of postoperative pain. Anesth Analg.

[34] Buvanendran A, Kroin JS, Della Valle CJ, Moric M, Tuman KJ (2016). Local Drug Infiltration Analgesia During Knee Surgery to Reduce Postoperative Pain in Rats. Reg Anesth Pain Med.

[35] McAlvin JB, Padera RF, Shankarappa SA, Reznor G, Kwon AH, Chiang HH (2014). Multivesicular liposomal bupivacaine at the sciatic nerve. Biomaterials.

[36] Zhao C, Liu A, Santamaria CM, Shomorony A, Ji T, Wei T (2019). Polymer-tetrodotoxin conjugates to induce prolonged duration local anesthesia with minimal toxicity. Nat Commun.

[37] Shackelford C, Long G, Wolf J, Okerberg C, Herbert R (2002). Qualitative and quantitative analysis of nonneoplastic lesions in toxicology studies. Toxicol Pathol.

[38] Fafaj A, Krpata DM, Petro CC, Prabhu AS, Rosenblatt S, Tastaldi L (2020). The Efficacy of Liposomal Bupivacaine On Postoperative Pain Following Abdominal Wall Reconstruction: A Randomized, Double-Blind, Placebo-Controlled Trial. Ann Surg.

[39] Meyer LA, Corzo C, Iniesta MD, Munsell M, Shi Q, Pitcher B (2021). A prospective randomized trial comparing liposomal bupivacaine vs standard bupivacaine wound infiltration in open gynecologic surgery on an enhanced recovery pathway. Am J Obstet Gynecol.

[40] Ni Q, Chen W, Tong L, Cao J, Ji C (2016). Preparation of novel biodegradable ropivacaine microspheres and evaluation of their efficacy in sciatic nerve block in mice. Drug Des Devel Ther.

[41] Zhang W, Xu W, Ning C, Li M, Zhao G, Jiang W (2018). Long-acting hydrogel/microsphere composite sequentially releases dexmedetomidine and bupivacaine for prolonged synergistic analgesia. Biomaterials.

[42] Wang J, Zhang L, Chi H, Wang S (2016). An alternative choice of lidocaine-loaded liposomes: lidocaine-loaded lipid-polymer hybrid nanoparticles for local anesthetic therapy. Drug Deliv.

[43] Chen Y, Tang C, Zhang J, Gong M, Su B, Qiu F (2015). Self-assembling surfactant-like peptide A6K as potential delivery system for hydrophobic drugs. Int J Nanomedicine.

[44] Wei W, Tang J, Hu L, Feng Y, Li H, Yin C (2021). Experimental anti-tumor effect of emodin in suspension - in situ hydrogels formed with self-assembling peptide. Drug Deliv.

[45] Shah S, Shah SA, Faisal S, Khan A, Ullah R, Ali N (2021). Engineering novel gold nanoparticles using Sageretia thea leaf extract and evaluation of their biological activities. Journal of Nanostructure in Chemistry.

[46] Abdullah Rahman Au, Faisal S Almostafa MM, Younis NS Yahya G (2023). Multifunctional Spirogyra-hyalina-Mediated Barium Oxide Nanoparticles (BaONPs): Synthesis and Applications. Molecules.

