# Supplementary material for: Size control of ropivacaine nano/micro-particles by soft-coating with peptide nanosheets for long-acting analgesia
Source: Theranostics. 2024 Apr 15;14(6):2637–55. doi: 10.7150/thno.93322 (PMC11024846; doi:10.7150/thno.93322)
Supplement: Supplementary file 1 — Supplementary materials and methods, figures and tables. [file thnov14p2637s1.pdf]

Size control of ropivacaine nano/micro-particles by soft-coating with peptide nanosheets for long-acting analgesia

Jing Liu[1,2], Weiwei Wu[1,2], Fei Peng[1,2], Deying Gong[1,2], Yi Kang[1,2], Yujun Zhang[1,2], Congyan Liu[2], Yuncheng Li[2], Guoyan Zhao[2], Feng Qiu[1,2,*], Wensheng Zhang[1,2,*]

[1] Department of Anesthesiology, West China Hospital, Sichuan University, China

[2] Laboratory of Anesthesia and Critical Care Medicine, National-Local Joint Engineering Research Centre of Translational Medicine of Anesthesiology, West China Hospital, Sichuan University, China

Corresponding authors

Email: fengqiu@scu.edu.cn, zhang_ws@scu.edu.cn

Supplementary Materials and Methods

Circular Dichroism (CD)

CD spectrum of AG was measured using a Chirascan Plus CD spectrophotometer (Applied Photophysics, UK) to analyze the secondary structure. AG at different pH was diluted to 0.1 mM with Milli-Q water. CD spectrum was recorded from 185 to 260 nm in a cuvette with a path length of 2 mm.

ThT-binding test

ThT-binding fluorescence was used to examine the ability of AG to self-assemble under different pH. Briefly, ThT stock solution (1 mM in H_2O) was added to different AG solutions to get a final ThT concentration of 10 μM. The mixture was pipetted into a quartz cuvette to be measured with a spectrofluorophotometer (Horiba iHR320). The excitation wavelength was 450 nm and the emission spectrum between 460 and 600 nm was recorded.

To determine the critical aggregation concentration (CAC) of AG at different pH, AG solution at different pH was diluted to different concentration ranging from 0.01 to 5 mM. ThT fluorescence of each sample was measured as described above, and for samples at each pH, the peak value at 495 nm was plotted against the peptide concentration. CAC value of each pH was determined as where the fluorescence signal at 495 nm began to raise drastically.

Pyrene fluorescence

Pyrene fluorescence test was performed to detect hydrophobic region in AG at different pH. Briefly, 1 μL of pyrene stock solution (2 mM in dimethyl sulphoxide) was mixed with 499 μL of 5 mM AG solution or Milli-Q water and incubated at room temperature for 2-5 min. The fluorescence spectra between 360-440 nm were recorded at the excitation wavelength of 336 nm. Obtained spectra were normalized to get a same value of the first peak (I1), and the ratio of I1 and I3 (the third peak) was calculated. The monomer/excimer fluorescence spectrum of pyrene@AG particles ranging from 360-600 nm was recorded at the same excitation wavelength.

Fourier transform infrared (FTIR) spectroscopy

Lyophilized powder of AG peptide, RH, RB or RB@AG particles were analyzed

by attenuated total internal reflectance FTIR (INVENIO R, Bruker, German). Spectra between 400-4000 cm^{-1} were collected with a resolution of 4 cm^{-1} and a scan count of 16.

Dynamic light scattering (DLS)

DLS experiments were performed to measure the size and zeta potential of the peptide and RB@AG particles. Zetasizer Nano ZS90 and Mastersizer 3000 (Malvern Panalytical, Malvern, UK) was used to measure size distribution of AG peptide and RB@AG particles, respectively, while zeta potential was measured by Zetasizer Nano ZS90. Each sample was measured for three times and averaged results were obtained.

Transmission electron microscopy (TEM) observation

AG solution (5 mM) at different pH was dropped onto the surface of a copper grid and incubated for 3.5 min, after which excess solution was blotted with filter paper. The sample was then negatively stained with 2% phosphotungstic acid for 1.5 min, and excess staining solution was blotted with filter paper. The copper grid was air-dried and observed with TEM (Tecnai G2 F20, FEI, USA).

Atomic force microscopy (AFM)

To observe the self-assembling structure of AG at different pH, 10-20 μL of 5 mM sample was dropped onto the surface of a freshly cleaved mica flake and air-dried. Then the mica flake was rinsed with water and air-dried. The sample was observed with AFM (SPM-9700HT, Shimadzu, Japan), and line profile analysis was performed to estimate the height of the nanostructures.

Scanning electron microscopy (SEM)

Suspension of pyrene, pyrene@AG, RB or RB@AG particles was spread onto a clean 5 mm × 5 mm glass slide and air-dried. The slide was glued to a sample holder and treated with gold spray for 180 sec to coat the sample. Then the slide was observed under SEM (EVO 10, ZEISS, German).

Fluorescence microscopy

ThT with the final concentration of 1 μM was added to pyrene@AG or RB@AG particles and incubated for half an hour at room temperature. Then the particles were

washed twice and resuspended in PB (10 mM, pH 7.4). Resuspended particles uniformly spread on the clean slide was air-dried and observed under fluorescence microscope (ZEISS, German).

Powder X-ray diffraction (XRD)

XRD experiment was carried out to analyze the crystal structure of lyophilized powder of AG peptide, RH, RB, pyrene, pyrene@AG or RB@AG particles. Parameters were set as followed: generator voltage 40 kV, tube current 40 mA, 2θ angular range 5-50°, angular reproducibility +/- 0.0001°, step size 0.0262606°, detector count matrix 256 × 256, pixel size 55 mm × 55 mm, resolution full half-peak width of 0.028°.

X-ray photoelectron spectroscopy (XPS)

XPS was used to analyze the chemical compositions of the AG peptide, RB, pyrene, pyrene@AG or RB@AG formulation. XPS high-resolution scans were collected using a Kratos Axis ULTRA X-ray photoelectron spectrometer (Kratos, UK). Measured spectra were acquired at a pass energy of 160 eV, and high-resolution scans were taken at a pass energy of 20 eV. The data were analyzed using CasaXPS software (Version 2.3.19PR1.0).

Sciatic nerve block (SNB) model

Rat SNB model was used to evaluate the anesthetic efficacy of different formulations. To start with, the rats were placed in right lateral recumbency after anesthetized with 2-4% isoflurane. Between the line of greater trochanter and ischial tuberosity, 0.2 mL of each formulation was injected over the left sciatic nerve using a syringe with 26 G needle.

At different time point after drug injection, a modified hotplate test was carried to evaluate the sensory block. Briefly, the rat was lifted so that its left hind paw was placed on the hot plate which was set at a temperature of 55 ± 1 °C, and the latency time for the rat to withdraw its paw (paw withdraw latency, PWL) was measured with a stopwatch. To prevent the rat from scald, the cutoff value was set to 12 sec. The latency time no less than 6 sec was defined as effective sensory block. The maximum possible effect (MPE) was calculated according to the following formula:

$$\text{MPE (\%)} = \frac{\text{PWL}_{\text{test}} - \text{PWL}_{\text{baseline}}}{\text{PWL}_{\text{cutoff}} - \text{PWL}_{\text{baseline}}} \times 100$$

PWL_{test} is the PWL value of test after drug administration, PWL_{cutoff} is 12 sec, $PWL_{baseline}$ is the PWL value of baseline.

At the same time, the motor block effect was assessed through the postural extensor thrust (PET) test which was measured as gram (g). The rat was held parallel to the electronic balance (model HZT-B5000, Huazhi Scientific Instrument Co., Ltd., China) with its left hind limb stomping on the balance and the value was recorded. It was defined as effective motor block when the PET value was lower than a half of the baseline.

Pharmacokinetic study

For evaluating changes of plasma ropivacaine concentration, rats were injected with different ropivacaine formulations as described in the SNB model. At different time point after injection, approximately 0.3 mL of blood were collected into heparinized tubes from the tail vein. Subsequently, the blood was centrifuged at 3500 rpm, 4 °C for 10 min. The obtained plasma was collected in a new tube and stored at -40 °C until analysis. The content of ropivacaine in plasma was measured using liquid chromatography-mass spectrometry. The corresponding pharmacokinetic parameters were calculated using the Drug and Statistics (DAS, version 3.3.0) noncompartmental model for analysis.

Total knee arthroplasty (TKA) model

To evaluate the analgesic efficacy of different formulations, TKA was used as a postoperative pain model. Briefly, the rat was kept supine after anesthetized by isoflurane. After shaved and sterilized with 75% ethanol, an incision about 1 cm long was made on the medial aspect of the left posterior knee. Patella tendon was exposed and pulled laterally to expose the lower femur and upper tibia. A hole with 1.2 mm width and 0.5 mm depth in each of the femur and tibia was made with an electronic drill. After applying pressure to stop bleeding, 10 μL of different formulation was injected into each hole. The incision was closed with 4-0 nylon suture after injection.

The locomotor activities of rats after TKA were evaluated through open field test, which reflected the postoperative pain indirectly. The day before experiment, each rat was placed in the center of the arena (100 × 100 × 40 cm) to acclimatize for 3-5 min. At 6, 24 and 48 h after the TKA surgery and drug administration, each rat was placed in the arena to test for 10 min. The travel distance was recorded by video-tracking

system (Smart version 3.0, Panlab, USA). The light condition was remained the same and the environment remained quiet during the entire procedure.

Cytotoxicity

PC12 and C2C12 cells purchased from the American Type Culture Collection (ATCC, USA) were used to evaluate the cytotoxicity of AG. PC 12 and C2C12 cells were cultured in RPMI 1640 medium with 15% fetal bovine serum (FBS) and DMEM with 10% FBS, respectively, both of which were supplemented with 1% penicillin and streptomycin. Culture condition was 37 °C in a humidified 5% CO_2 atmosphere. PC 12 and C2C12 cells were seeded in 96-well plates at a density of 1×10^4 and 6×10^3 cell/well, respectively. After overnight incubation, cells were cultured in fresh medium containing AG peptide at different concentration. Cell viability was evaluated using CCK8 kit (MedChemExpress, USA) after exposure to AG for 24 h. The OD value at 450 nm was measured using microplate reader. The relative cell viability was calculated according to the following equation:

$$\text{Survival rate (\%)} = \frac{OD_{test} - OD_{medium}}{OD_{control} - OD_{medium}} \times 100$$

The OD_{test} is the OD value of cells received test drug, the OD_{medium} is the OD value of cells without any treatment, the $OD_{control}$ is the OD value of cells received equal volume PBS.

Supplementary Figures and Tables

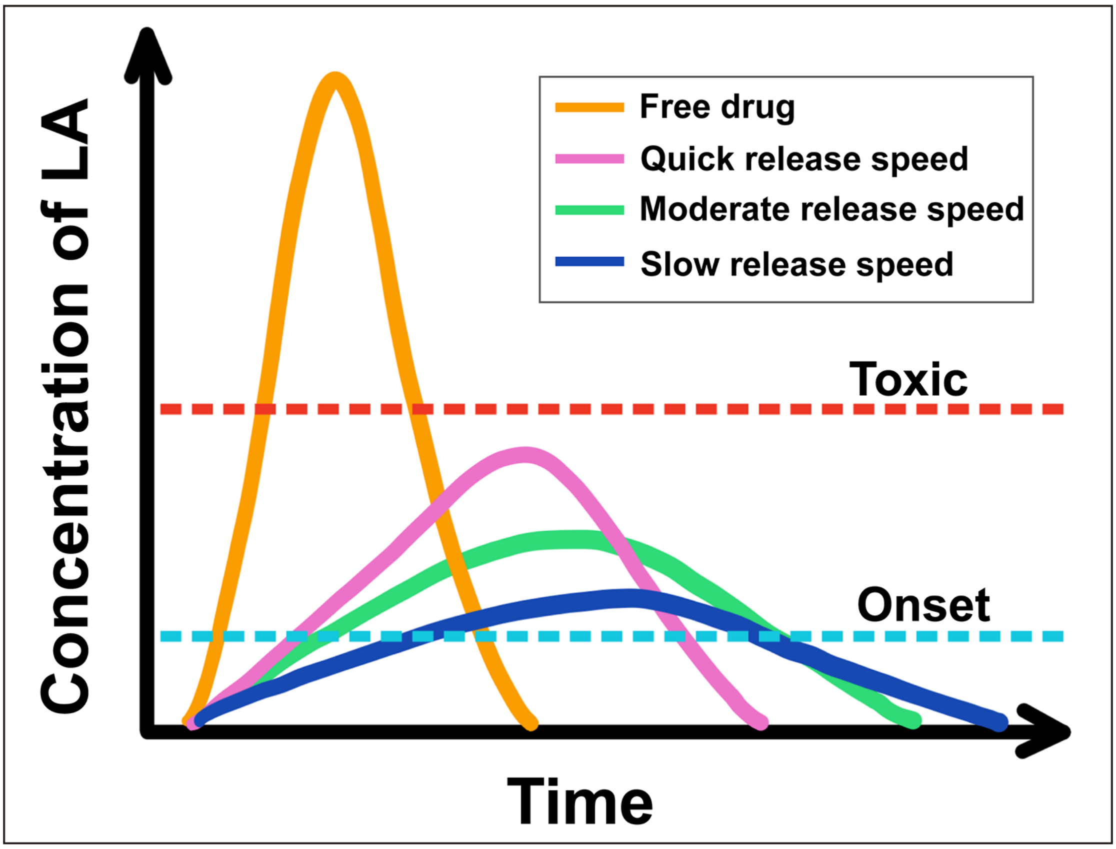

Figure S1. Illustration of optimal release speed for long-acting anesthesia considering the effective and toxic thresholds.

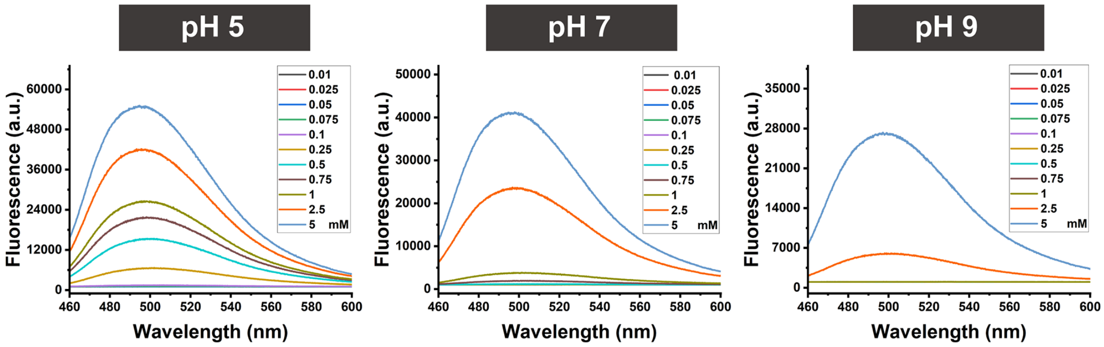

Figure S2. ThT-binding fluorescence spectra of AG at different pH and different concentration.

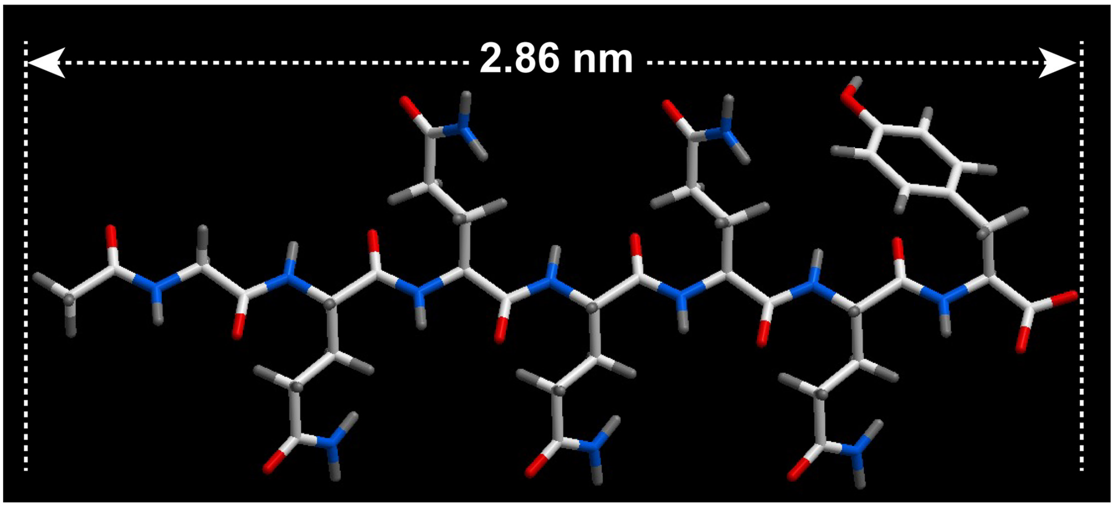

Figure S3. Molecule model of AG peptide. The model was generated by the ICM-Pro software package (MolSoft LLC, San Diego, Calif.), and estimated length of the peptide monomer was 2.86 nm.

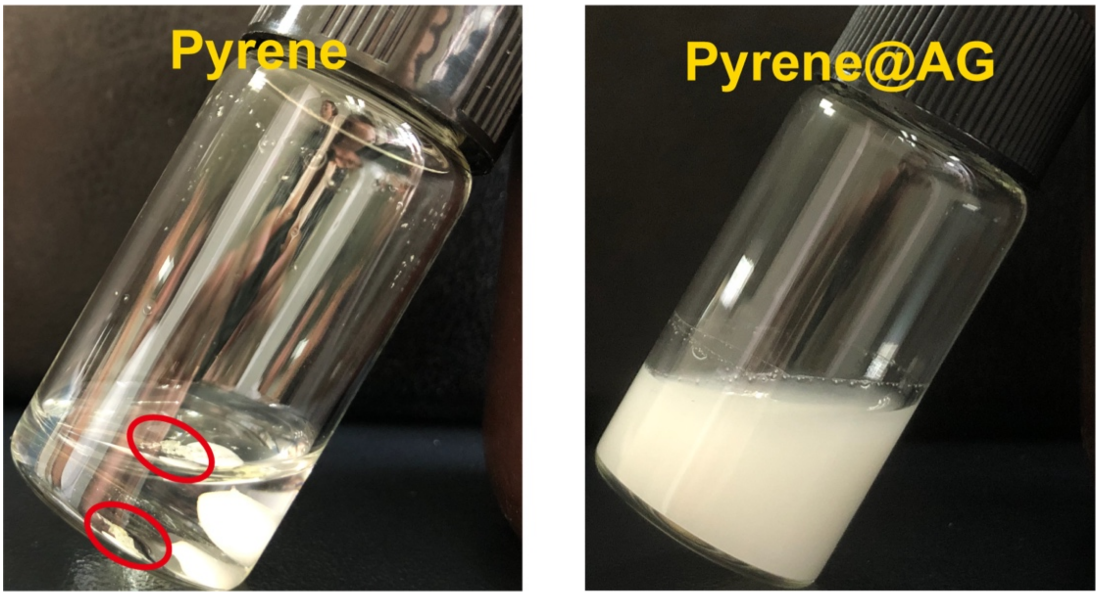

Figure S4. Photographs of pyrene dispersed in PBS or AG peptide. Red circles marked big pyrene crystals that could not well-dispersed in PBS, while a milky suspension was obtained in AG.

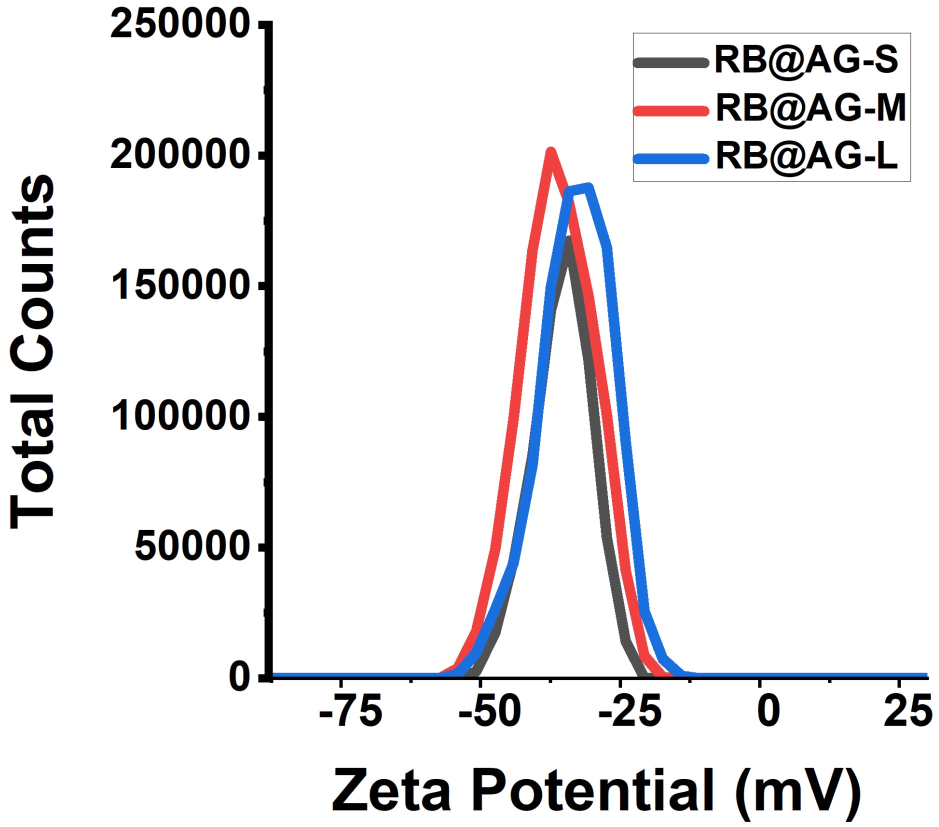

Figure S5. Zeta potential of RB@AG particles with different size.

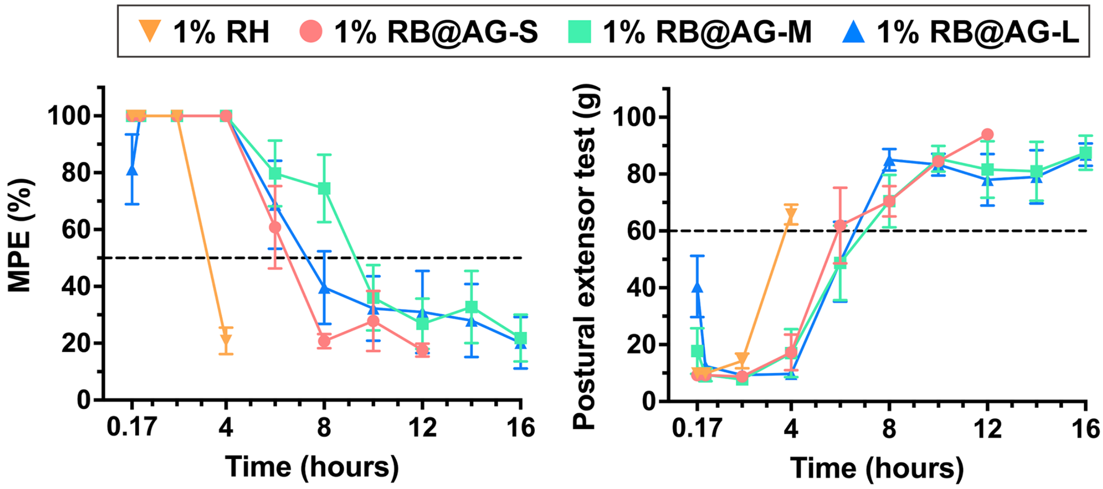

Figure S6. MPE and PET value of RB@AG particles with different size. Left panel: MPE of sensory nerve block. The dotted line shows MPE value of 50%, MPE values more than which are defined as effective sensory block (n = 8). Right panel: PET value of motor nerve block. The dotted line indicated PET value of 60 g, values above which are defined as motor block recovery (n = 8).

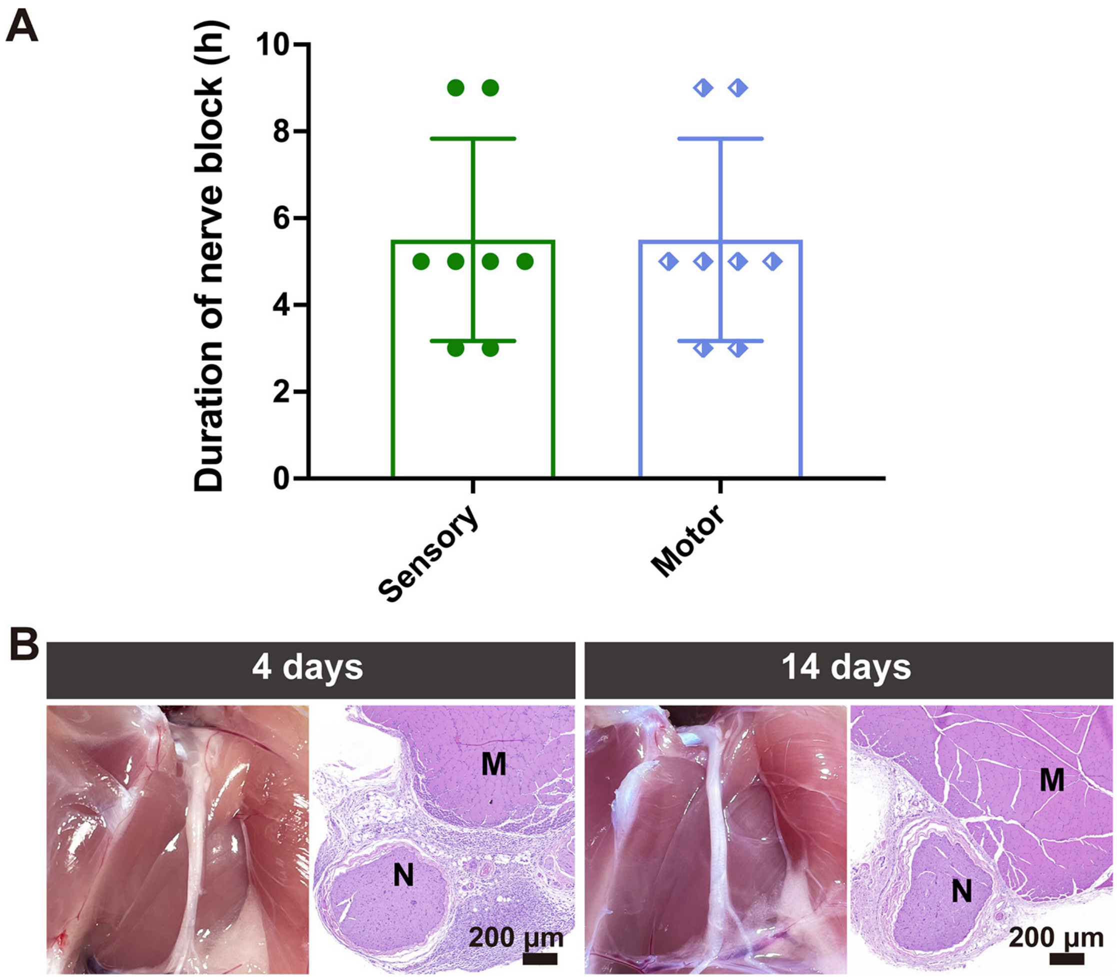

Figure S7. Anesthetic efficacy and local toxicity of 1.33% BUP@LS. (A) Nerve block efficacy of 1.33% BUP@LS in rat SNB model (n = 8). (B) Representative photographs of injection site and microscope images of HE stained sections of sciatic nerve and adjacent muscle after injected with 1.33% BUP@LS.

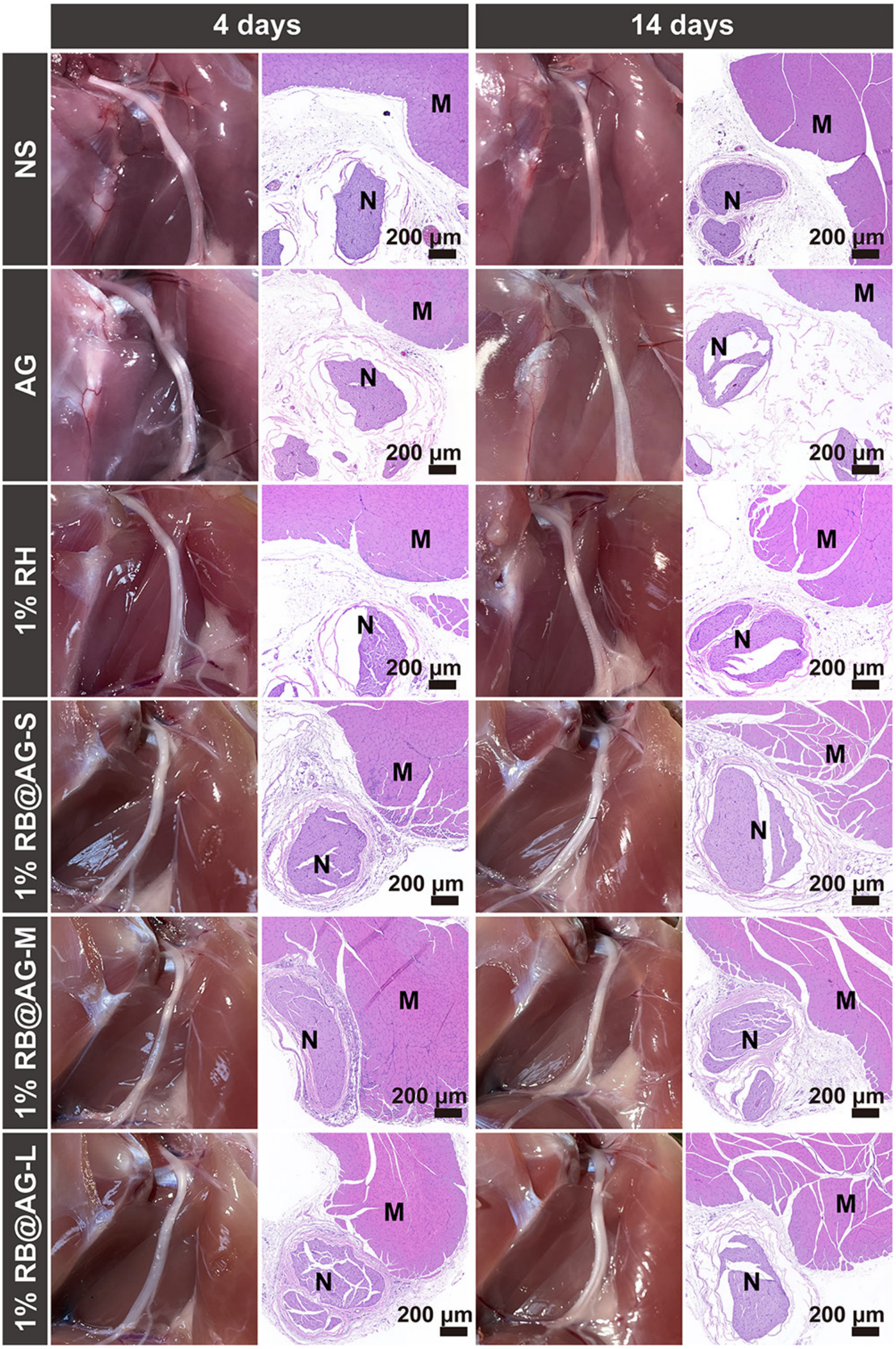

Figure S8. Representative photographs of injection site and microscope images of HE stained sections of sciatic nerve and adjacent muscle after injected with NS, AG, 1% RH or 1% RB@AG formulations with different size.

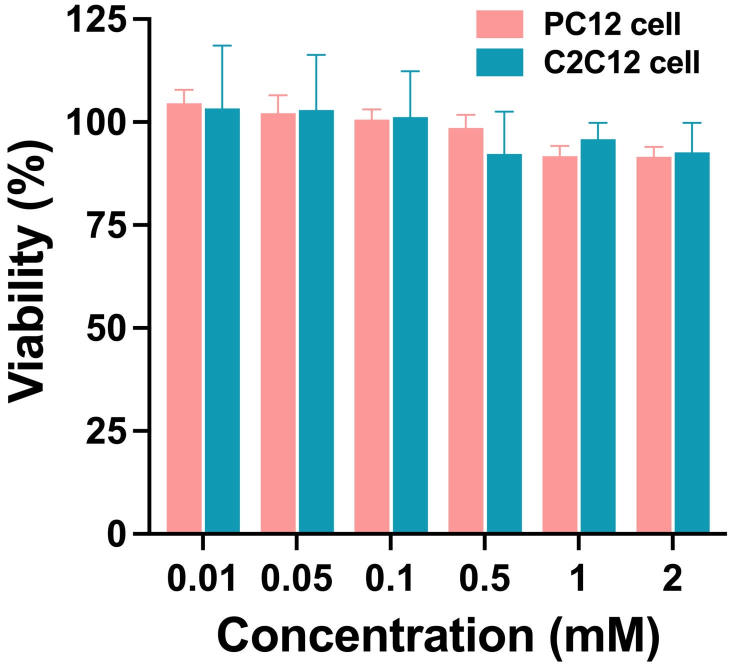

Figure S9. Cytotoxicity of AG in PC12 and C2C12 cells.

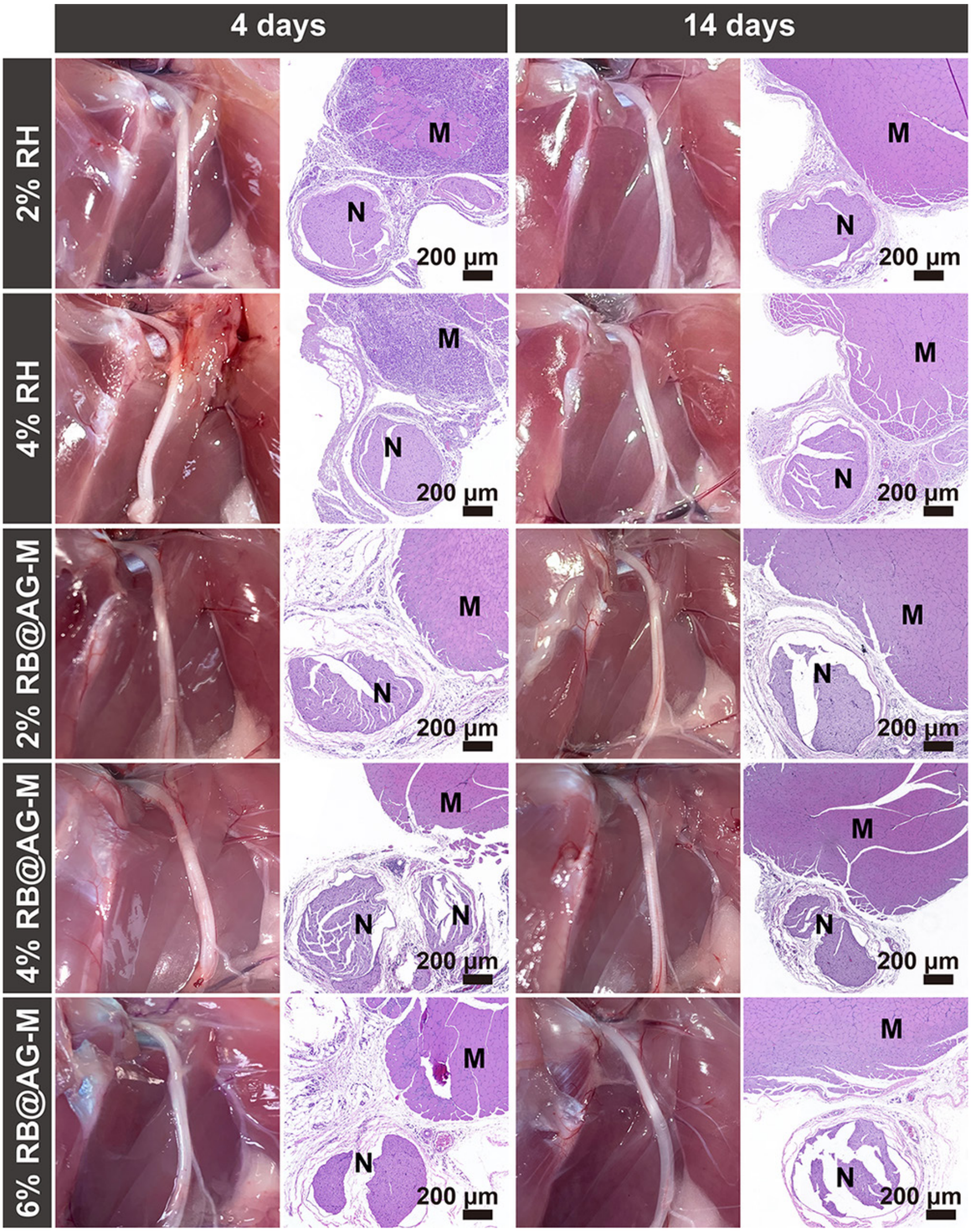

Figure S10. Representative photographs of injection site and microscope images of HE stained sections of sciatic nerve and adjacent muscle after injected with RH and RB@AG-M at different concentration.

Table S1. Pharmacokinetics parameters of different 1% ropivacaine formulations.

Group	C_{max} (μg/L)	T_{max} (h)	$MRT_{(0-t)}$ (h)	$MRT_{(0-\infty)}$ (h)	$AUC_{(0-t)}$ (μg/L*h)	$AUC_{(0-\infty)}$ (μg/L*h)
1% RH	687 ± 129	1.17 ± 0.41	1.97 ± 0.13	2.02 ± 0.16	1817 ± 251	1830 ± 253
1% RB@AG-S	241 ± 53*	2.08 ± 1.11*	6.07 ± 0.79*	6.68 ± 0.97	1771 ± 174	1814 ± 167
1% RB@AG-M	175 ± 25*#	4.00 ± 2.19*	7.29 ± 0.58*#	8.12 ± 1.12	1697 ± 215	1755 ± 211
1% RB@AG-L	119 ± 43*#&	5.67 ± 2.66*#	9.03 ± 0.48*#&	11.17 ± 2.13	1435 ± 314	1581 ± 409

Data were expressed as mean ± standard deviation (SD), n = 6. *, compared with 1% RH, $p < 0.05$, C_{max} and $MRT_{(0-t)}$ was analyzed using one-way ANOVA followed by LSD and Dunnett T3 post-hoc test, respectively, and T_{max} was analyzed using non-parametric test followed by Bonferroni post-hoc test. #, compared with 1% RB@AG-S, $p < 0.05$; &, compared with 1% RB@AG-M, $p < 0.05$; one-way ANOVA followed by LSD post-hoc test.

Table S2. Pharmacokinetics parameters of different ropivacaine formulations with different concentration.

Group	C_{max} (μg/L)	T_{max} (h)	$MRT_{(0-t)}$ (h)	$MRT_{(0-\infty)}$ (h)	$AUC_{(0-t)}$ (μg/L*h)	$AUC_{(0-\infty)}$ (μg/L*h)
2% RH	898 ± 144	0.83 ± 0.26	2.00 ± 0.25	2.06 ± 0.25	2593 ± 229	2613 ± 234
4% RH	1812 ± 395$^{\#}$	0.67 ± 0.26	2.17 ± 0.48	2.31 ± 0.61	5630 ± 512	5745 ± 587
2% RB@AG-M	176 ± 47*	8.00 ± 3.10*	10.03 ± 1.09*	10.60 ± 1.71	2667 ± 266	2718 ± 248
4% RB@AG-M	298 ± 49$^{*\&}$	8.50 ± 1.76*	13.51 ± 2.85*	14.84 ± 4.17	5751± 538	5906 ± 601
6% RB@AG-M	323 ± 69$^{*\&}$	10.67 ± 1.63*	22.79 ± 3.25$^{*\&\$}$	27.07 ± 4.67	9066 ± 1135	9528 ± 1056

Data were expressed as mean ± SD, n = 6. #, compared with 2% RH, $p < 0.05$; *, compared with 2%RH and 4% RH, $p < 0.01$; C_{max} and $MRT_{(0-t)}$ was analyzed using one-way ANOVA followed by Dunnett T3 post-hoc test, and T_{max} was analyzed using non-parametric test followed by Bonferroni post-hoc test. &, compared with 2% RB@AG-M, $p < 0.05$; $, compared with 4% RB@AG-M, $p < 0.05$; C_{max} and $MRT_{(0-t)}$ was analyzed using one-way ANOVA followed by LSD post-hoc test, and T_{max} was analyzed using non-parametric test followed by Bonferroni post-hoc test.

Table S3. Comparison of local injury of rats after injected with different ropivacaine formulations in SNB model.

Formulation	Inflammation score (0-4)		Myotoxicity score (0-6)		Axonal degeneration score (0-4)	
	Day 4	Day 14	Day 4	Day 14	Day 4	Day 14
**1% RH**	1.0 (0.25-1.0)	0 (0-0)	3.0 (0.75-3.0)	1.0 (1.0-1.0)	0 (0-0)	0 (0-0)
**2% RH**	2.5 (2.0-3.0)	0.5 (0-1.0)	4.5 (4.0-5.0)	2.0 (1.25-2.0)	1.0 (0-2.0)	1.5 (1.0-2.0)
P value (versus 1% RH)	0.042	0.453	0.042	0.215	0.302	0.056
**4% RH**	2.5 (1.0-3.0)	0.5 (0-1.0)	4.5 (4.0-5.0)	2.0 (2.0-2.75)	0.5 (0-1.0)	1.0 (0-2.0)
P value (versus 1% RH)	0.042	0.453	0.042	0.016	0.723	0.402
P value (versus 2% RH)	1.000	1.000	1.000	0.978	1.000	1.000
**2% RB@AG-M**	1.0 (0.25-1.0)	0.5 (0-1.0)	2.0 (0.25-3.0)	1.0 (1.0-1.0)	0 (0-0.75)	0 (0-0)
P value (versus 1% RH)	1.000	0.683	1.000	1.000	1.000	1.000
P value (versus 2% RH)	0.017	1.000	0.019	0.040	0.317	0.013
P value (versus 4% RB@AG)	1.000	1.000	1.000	0.301	1.000	1.000
**4% RB@AG-M**	1.0 (1.0-1.0)	0 (0-0.75)	1.5 (0-3.0)	0 (0-0.75)	0 (0-0.75)	0 (0-0)
P value (versus 1% RH)	1.000	1.000	1.000	0.301	1.000	1.000
P value (versus 4% RH)	0.013	0.495	0.018	0.015	0.495	0.127
P value (versus 6% RB@AG)	0.160	1.000	1.000	1.000	1.000	0.944
**6% RB@AG-M**	0 (0-0.75)	0 (0-0.75)	0 (0-2.25)	0.5 (0-1-1.75)	0 (0-0.75)	0 (0-0.75)
P value (versus 1% RH)	0.838	1.000	0.939	1.000	1.000	0.944
P value (versus 2% RB@AG)	0.838	1.000	1.000	1.000	1.000	0.944

Data were presented as median with 25th and 75th percentiles; n = 4 for all groups. The difference of local injury between 1%, 2% and 4% RH of that between 2%, 4% and 6% RB@AG-M formulations, was compared using nonparametric tests followed by Bonferroni post-hoc test.

Table S4. Comparison of local injury of rats after injected with 1% RH or 1.33% BUP@LS in SNB model.

Formulation	Inflammation score (0-4)		Myotoxicity score (0-6)		Axonal degeneration score (0-4)	
	Day 4	Day 14	Day 4	Day 14	Day 4	Day 14
1% RH	1.0 (0.25-1.0)	0 (0-0)	3.0 (0.75-3.0)	1.0 (1.0-1.0)	0 (0-0)	0 (0-0)
1.33% BUP@LS	2.0 (2.0, 2.0)	1.0 (0.25-1.0)	3.5 (3.0-4.0)	2.0 (2.0, 2.0)	0 (0-0)	0 (0-1.5)
P value	0.011	0.040	0.096	0.008	1.000	0.317

Data were presented as median with 25th and 75th percentiles; n = 4 for all groups. P values are for the comparison of the tissue reaction of 1.33% BUP@LS to that of 1% RH (Manne-Whitney U test).

Table S5. The free ropivacaine concentration and EE of 6% RB@AG-M formulation after stored at 4 ℃ for different time.

Time (week)	Con. of free ropivacaine (mg/mL)	EE (%)
0	1.45 ± 0.04	97.58 ± 0.07
1	1.29 ± 0.02	97.85 ± 0.03
2	1.46 ± 0.04	97.56 ± 0.06
3	1.62 ± 0.04	97.30 ± 0.06
5	1.54 ± 0.03	97.44 ± 0.06
8	1.22 ± 0.03	97.97 ± 0.05
